# Transcriptome analysis of clock disrupted cancer cells reveals differential alternative splicing of cancer hallmarks genes

**DOI:** 10.1038/s41540-022-00225-w

**Published:** 2022-05-12

**Authors:** Deeksha Malhan, Alireza Basti, Angela Relógio

**Affiliations:** 1 Institute for Theoretical Biology (ITB), Charité – Universitätsmedizin Berlin, corporate member of Freie Universität Berlin, Humboldt – Universität zu Berlin, and Berlin Institute of Health, Berlin, 10117 Germany; 2 Molecular Cancer Research Center (MKFZ), Medical Department of Hematology, Oncology, and Tumor Immunology, Charité – Universitätsmedizin Berlin, corporate member of Freie Universität Berlin Humboldt – Universität zu Berlin, and Berlin Institute of Health, Berlin, 10117 Germany; 3 Institute for Systems Medicine, Faculty of Human Medicine, MSH Medical School Hamburg, Hamburg, 20457 Germany

**Keywords:** Cancer, Computational biology and bioinformatics

## Abstract

Emerging evidence points towards a regulatory role of the circadian clock in alternative splicing (AS). Whether alterations in core-clock components may contribute to differential AS events is largely unknown. To address this, we carried out a computational analysis on recently generated time-series RNA-seq datasets from three core-clock knockout (KO) genes (*ARNTL*, *NR1D1*, *PER2*) and WT of a colorectal cancer (CRC) cell line, and time-series RNA-seq datasets for additional CRC and Hodgkin’s lymphoma (HL) cells, murine WT, *Arntl* KO, and *Nr1d1/2* KO, and murine SCN WT tissue. The deletion of individual core-clock genes resulted in the loss of circadian expression in crucial spliceosome components such as *SF3A1* (in *ARNTL*^KO^), *SNW1* (in *NR1D1*^KO^), and *HNRNPC* (in *PER2*^KO^), which led to a differential pattern of KO-specific AS events. All HCT116^KO^ cells showed a rhythmicity loss of a crucial spliceosome gene *U2AF1*, which was also not rhythmic in higher progression stage CRC and HL cancer cells. AS analysis revealed an increase in alternative first exon events specific to *PER2* and *NR1D1* KO in HCT116 cells, and a KO-specific change in expression and rhythmicity pattern of AS transcripts related to cancer hallmarks genes including *FGFR2* in HCT116_*ARNTL*^KO^, *CD44* in HCT116_*NR1D1*^KO^, and *MET* in HCT116_*PER2*^KO^. KO-specific changes in rhythmic properties of known spliced variants of these genes (e.g. *FGFR2* IIIb/*FGFR2* IIIc) correlated with epithelial-mesenchymal-transition signalling. Altogether, our bioinformatic analysis highlights a role for the circadian clock in the regulation of AS, and reveals a potential impact of clock disruption in aberrant splicing in cancer hallmark genes.

## Introduction

Alternative splicing (AS) is a central event in transcriptional regulation leading to distinct mRNAs being generated from a single gene, and is thus a major contributor for protein diversity^1,2^. Approximately 95% of human genes undergo AS in a tissue type-specific manner^3^, and AS resulting products lead to the production of proteins involved in numerous biological processes including cell cycle^4^ and metabolism^5^. Splicing factors (SFs) mark the splice sites, facilitate spliceosome assembly during the splicing process, and can regulate AS decisions and thus the resulting splicing product(s)^6^. Dysregulations in splicing account for the production of non-functional, or with the incorrect function, protein products with an impact on the onset and progression of several diseases among which cancer^7–12^. A systematic evaluation of 11 different tumour types revealed a subset of transcript isoform switches associated with gain and/or loss of protein domains, which are associated with the hallmarks of cancer^13^. Known examples of AS failures related to cancer include the aberrant splicing in the Vascular Epithelial Growth Factor (*VEGF*), essential for angiogenesis, resulting in one transcript isoform with anti-angiogenic properties in cancer^14^. Another example is *CD44*, which encodes for a cell-surface protein and can act as both tumour-suppressing, as well as invasiveness-promoting molecule, depending on its different splice variants^15^. In addition, alterations in the expression of several SFs can impact the splicing of their target genes, for e.g., the differential expression of the splicing factor Serine and Arginine Rich Splicing Factor 6 (*SRSF6*) affects the splicing of several oncogenes including the Insulin Receptor (*INSR*), MAPK Interacting Serine/Threonine Kinase 2 (*MKNK2*) and Discs Large MAGUK Scaffold Protein 1 (*DLG1*) leading to the production of oncogenic isoforms, or to the reduction of the tumour-suppressing isoforms of those genes^16^. Also, aberrant AS in other members of the SR splicing factor and SR-like families, including *SRSF4* and Transformer 2 Beta Homolog (*TRA2β*) have been shown to regulate cancer proliferation and metastasis by affecting the splicing of genes involved in epithelial-mesenchymal transition (EMT, e.g., *CD44*) in breast cancer^17^. Mounting evidence suggests as well an association between AS regulation and the circadian clock in various organisms in particular in mammals^18,19^.

Circadian rhythms are driven by an endogenous oscillator with an approximate period of 24 h and regulate the timing of cellular processes in most organisms^20^. These rhythms are generated by a Transcriptional-Translational Feedback Loop (TTFL) in which Brain and muscle ARNT-Like 1 (BMAL1), also known as ARNTL forms a heterodimer with Circadian Locomoter Output Cycles Kaput (CLOCK) and regulates the transcriptional activation of Period 1/2/3 (PER 1/2/3), and Cryptochrome 1/2 (CRY 1/2), which inhibit BMAL1-CLOCK-mediated transcription^21^. In addition, Nuclear Receptor Subfamily 1 Group D Member 1, 2 (NR1D1/2) and RAR Related Orphan Receptor A, B, C (RORA/B/C) regulate *BMAL1* transcription and contribute to the fine-tuning of its expression. The circadian clock regulates the expression of genes and proteins related to several cancer hallmarks like cell proliferation, metabolism, and DNA damage^22^. Disruption of circadian rhythms is associated with increased risk of cancer, including prostrate, breast, and colon cancer^23^.

Recent work points to a complex cross-talk between the circadian clock and AS events underlying different cancer types^24–31^. Changes in amplitude and peak phase of SFs including hnRNPs and RBM proteins in colorectal cancer (CRC) cells were correlated with altered splicing outcome of target genes like *VEGFA* and *CD44*^24^. A later study in the same cell lines reported rhythmic alternative splicing events of cancer-related genes like Cold inducible RNA binding protein (*CIRBP*) and Poly(RC) Binding Protein 2 (*PCBP2*)^25^. Clock-mediated alternative splicing events in the cell differentiation marker Interferon Regulatory Factor 4 (*IRF4*) were reported in an in vitro model of Hodgkin lymphoma (HL)^26^. In the same HL cell lines, clock-mediated changes in the expression of transcript isoforms were reported in elements associated with the tumour necrosis factor (TNF) pathway, which controls cell proliferation, migration, and apoptosis^27^. Rhythmic alternative splicing of the splicing factor *U2AF26* controls the stability of Per1 in mice^30^. A splice variant of human *BMAL1* (*hBMAL1a*) is known to act opposite to its canonical isoform (*hBMAL1b*) and functions as a negative regulator of the circadian clock^31^. However, the impact in alternative splicing events resulting from clock alterations in cancer remains unexplored.

In this study, we analysed time-course RNA-seq datasets generated from three HCT116 core-core knockouts (*ARNTL, PER2, NR1D1*) and wild type (WT) cells, to investigate the impact of clock disruption on AS events in CRC cell lines. In addition, we retrieved publicly available time-course RNA-seq datasets from human CRC cell lines (SW480, SW620), HL cell lines (HDMYZ, L1236), and mouse *Arntl* KO, *Nr1d1/2* KO, and SCN (Suprachiasmatic nucleus) WT tissue. Our study revealed that deletion of core-clock genes (*ARNTL*, *PER2* or *NR1D1)* altered the circadian rhythmic pattern of SFs, as well as the resulting AS events in a KO-specific manner. We observed a common rhythmicity loss of crucial SFs such as U2 Small Nuclear RNA Auxiliary Factor 1(*U2AF1*) in all three HCT116^KO^, as well as in SW620, and L1236 cells. Moreover, we found a phase shift in circadian transcripts of SFs such as Receptor of Activated C Kinase 1 (*RACK1*), Heterogeneous nuclear ribonucleoprotein D (*HNRNPD*) involved in pre-mRNA processing and Serine and Arginine rich splicing factor 5 (*SRSF5*), component of the spliceosome machinery, in HCT116_*ARNTL*^KO^, HCT116_*NR1D1*^KO^, and HCT116_*PER2*^KO^ vs. WT, respectively. This led to a distinct pattern of AS events (specifically in gain and loss of splicing events) in HCT116^KO^ datasets vs. HCT116_WT. Differential rhythmicity analysis revealed that the majority of phase shifted transcript pairs consisted of protein-coding biotypes. In HCT116^KO^ cells, genes with phase-shifted transcript pairs were related to apoptotic signalling (HCT116_*ARNTL*^KO^), macrophage proliferation (HCT116_*NR1D1*^KO^), and negative regulation of chromatin organization (HCT116_*PER2*^KO^). Moreover, we identified differentially rhythmic transcript isoform pairs of target genes associated with cancer hallmarks. These included Casein Kinase 2 Alpha 1 (*CSNK2A1*), related to invasion, metastasis, tumour proliferation, in HCT116_WT and HCT116^KO^. We further identified phase shifted transcript pairs from cancer hallmarks-related genes e.g., transcripts pairs from MET Proto Oncogene (*MET*), which controls several cancer hallmarks, in HCT116_*PER2*^KO^. Finally, we observed KO-specific altered expression and rhythmicity pattern of transcripts associated with cancer hallmarks related genes such as in Fibroblast Growth Factor Receptor 2 (*FGFR2*), which promotes tumour progression seen in HCT116_*ARNTL*^KO^, Caspase 8 (*CASP8*) which regulates extrinsic apoptotic pathway seen in HCT116_*NR1D1*^KO^, and HRas proto-oncogene, GTPase (*HRAS*) involved in cell proliferation, growth and apoptosis detected in HCT116_*PER2*^KO^. The evaluation of coding exon sequences for selected cancer hallmarks revealed the altered rhythmicity in spliced variants associated with cancer progression such as skipped exon 14 variant of *MET*. Our findings point to a direct impact of circadian clock disruption in AS events related to cancer hallmarks, which may support cancer onset or progression.

## Results

### Rhythmicity analysis of mammalian RNA-seq datasets showed a KO-specific effect in the circadian phenotype both at the gene and at the transcript levels

**Fig. 1:**
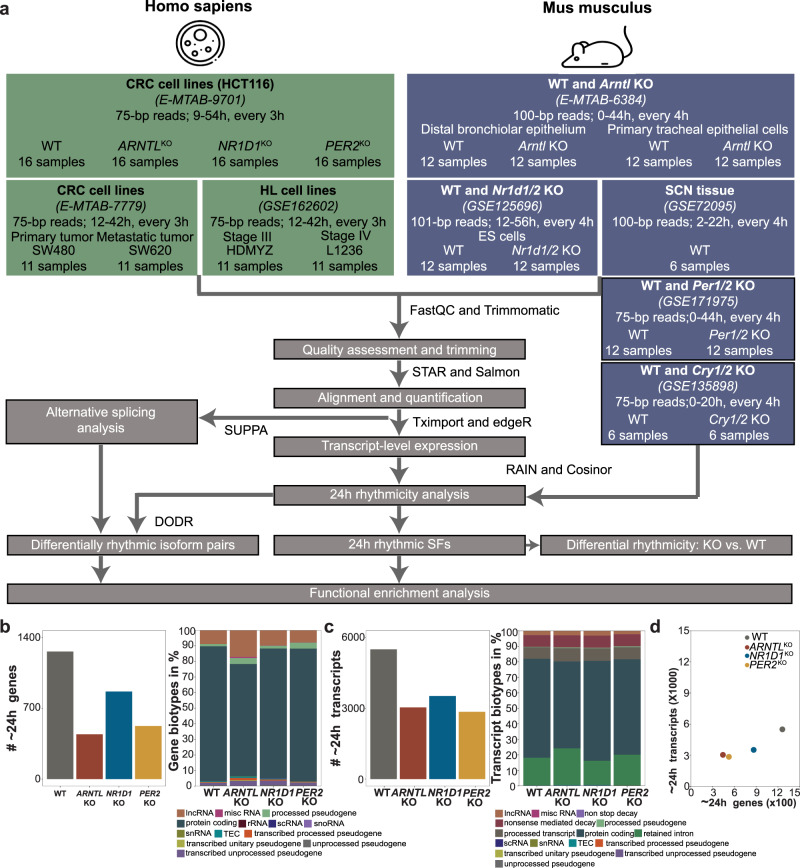
RNA-seq data analysis showed differences in the circadian phenotype between gene- and transcript expression values. **a** Schematic representation of the pipeline for RNA-seq data analysis. The input data consisted of 222 samples from eight mammalian time-course RNA-seq data. Murine datasets from *Per1/2* KO (GSE171975) and *Cry1/2* KO (GSE135898) were only analysed at the gene level. **b**–**d** Classification of 24 h rhythmic features in HCT116 datasets at (**b**) gene level and (**c**) transcript level showed a decline in the total number of circadian expressed genes/transcript-sets and changes in their biotypes due to core-clock disruption. **d** Scatter plot represents each of the HCT116 WT and KO cells according to the total number of 24 h rhythmic genes (*x*-axis) and the total number of 24 h rhythmic transcripts (*y*-axis).

In mammals, many different biological processes like metabolism or the immune system are regulated by the endogenous circadian clock^32^. To investigate the effect of clock disruption on the circadian phenotype (circa 24 h rhythmicity) and the subsequent effects in biological processes, we analysed recent datasets from our group for three core-clock knockout (KO) cell lines (HCT116_*ARNTL*^*KO*^, HCT116_*NR1D1*^*KO*^, and HCT116_*PER2*^*KO*^) and compared their gene expression pattern to the corresponding HCT116_WT (Fig. 1a, Supplementary Fig. 1d). Previous in vitro and in vivo studies showed an effect of *ARNTL*, *PER2* and *NR1D1* KO on the rhythmic expression of core-clock genes (mostly via bioluminescence recordings, or mouse studies)^33,34^, which are in agreement with our findings regarding the loss (*ARNTL* KO) and period alterations (*PER2* and *NR1D1* KO) in the respective oscillation profiles. Although, it is important to notice that the assessment of rhythmicity via in vitro bioluminescence measurements of *ARNTL* promoter activity, or via in vivo analysis of movement in mice, is not directly comparable with RNA-seq time course measurements.

To pinpoint specific changes due to the KO of core-clock genes, we additionally gathered publicly available time-course RNA-seq datasets for human CRC cell lines (SW480: derived from the primary tumour, SW620: derived from a metastasis site, from the same patient), HL cell lines (HDMYZ: stage III, L1236: stage IV), murine WT and *Arntl* KO, murine WT and *Nr1d1/2* KO, murine SCN tissue, murine WT and *Cry1/2* KO, and murine WT and *Per1/2* KO, resulting in a total of 222 samples (Fig. 1a). To examine if the rhythmicity pattern varies when analysed at the transcript level vs gene level, we extracted 24 h rhythmic sets both at gene and transcript levels in six-mammalian time course RNA-seq datasets (Fig. 1b–d, Supplementary Figs. 1–3). A higher number of 24 h rhythmic features were found at transcript level vs gene level in all datasets. The number of circadian (~24 h period) expressed genes and transcripts decreased in all three HCT116^KO^ vs. WT (Fig. 1b–d). At the gene level, we identified 1261, 443, 866 and 525 circadian gene sets in HCT116_WT, HCT116_*ARNTL*^KO^, HCT116_*NR1D1*^KO^, and HCT116_*PER2*^KO^, respectively (Fig. 1b**;** left panel). We observed KO-specific loss in circadian expression of crucial genes such as Keratin 8 (*KRT8*), which maintains cellular structural integrity, Transmembrane and Coiled-Coil Domain Family 1 (*TMCC1*), which promotes endoplasmic reticulum-associated fission, and HECT and RLD Domain Containing E3 Ubiquitin Protein Ligase 3 (*HERC3*), which enables protein-transferase activity in HCT116_*ARNTL*^KO^, HCT116_*NR1D1*^KO^, and in HCT116_*PER2*^KO^, respectively. At transcript level, we observed 5499 (corresponding to 4063 genes), 3039 (2485 genes), 3519 (2899 genes), 2850 (2345 genes) circadian transcripts in HCT116_WT, HCT116_*ARNTL*^KO^, HCT116_*NR1D1*^KO^, and HCT116_*PER2*^KO^, respectively (Fig. 1c; left panel). Among KO-specific loss of circadian expressed transcripts, we saw a loss of circadian transcript expression of C-Terminal Binding Protein 1 (*CTBP1*), which is a transcriptional repressor, *HRAS* an oncogene involved in signal transduction pathways, and Calmodulin 1 (*CALM1*), which controls several proteins including enzymes and ion channels, through calcium-binding. All these genes modulate cellular proliferation in HCT116_*ARNTL*^KO^, HCT116_*NR1D1*^KO^, and in HCT116_*PER2*^KO^, respectively.

Moreover, differences in the percentage of biotypes (e.g., protein-coding biotype) were also observed between circadian gene and transcript sets (Fig. 1b, c; right panel). For example, 1089 genes (~86%) and 3509 transcripts (~63%) with protein-coding biotype were circadian expressed in HCT116_WT. The comparison of rhythmicity at gene and transcript levels in HCT116 datasets showed a roughly linear relationship between the number of circadian genes and circadian transcripts (Fig. 1d). Similar differences in gene- and transcript-level rhythmicity phenotype were also seen in other human CRC cell lines (SW480; SW620), HL cell lines (HDMYZ; L1236), murine WT and *Arntl* KO, murine WT and *Nr1d1/2* KO, and murine SCN WT tissue datasets (Supplementary Figs. 2, 3). Within the set of circadian rhythmic genes, 846 genes (~67%) in HCT116_WT, 192 genes (~43%) in HCT116_*ARNTL*^KO^, 521 genes (~60%) in HCT116_*NR1D1*^KO^, and 274 genes (~52%) in HCT116_*PER2*^KO^ were rhythmic both at gene and transcript levels (Supplementary Fig. 1a). We further analysed circadian discrepancies between the expression pattern of some genes and their corresponding transcripts, for e.g., *HIF1A* was found to be circadian expressed whereas its transcripts were arrhythmic in HCT116_WT. Here, the summarization of arrhythmic transcripts resulted in a rhythmic gene. Minichromosome Maintenance Complex Component 2 (*MCM2*) was rhythmic both at gene and transcript levels in HCT116_WT. In contrast, Interleukin 18 (*IL18*) was identified as arrhythmic at the gene level whereas its transcripts were circadian expressed in HCT116_WT. In this case, the summarization of rhythmic transcripts resulted in an arrhythmic gene (Supplementary Fig. 1a; lower panel). Further, we analysed the changes in the number of transcripts per expressed gene and the number of ~24 h transcripts per gene in HCT116^KO^ vs. WT (Supplementary Fig. 1b, c). The number of expressed transcripts per gene showed variation in all three HCT116^KO^ vs. WT. For instance, 28 transcripts of Heterogeneous Nuclear Ribonucleoprotein H1 (*HNRNPH1*), an RNA binding protein, were expressed in HCT116_WT. Whereas, 25 transcripts from *HNRNPH1* were expressed in HCT116_*ARNTL*^KO^, 21 transcripts in HCT116_*NR1D1*^KO^, and 24 transcripts in HCT116_*PER2*^KO^. Out of the expressed transcripts of *HNRNPH1*, 5 transcripts in HCT116_WT and 1 transcript in HCT116_*PER2*^KO^ were circadian expressed. Altogether, our analysis confirmed previous findings from our group^25^, and showed that indeed a transcript-level analysis can provide new results that an be masked by gene-level analysis. Moreover, our analysis also shows that the KO of core-clock genes altered the ratio, as well as the circadian rhythmicity expression pattern of genes and transcripts in a KO-specific manner. Hence, we focused on transcript level in our subsequent analysis.

### Alterations in core-clock elements influenced the rhythmic expression of splicing factors

**Fig. 2:**
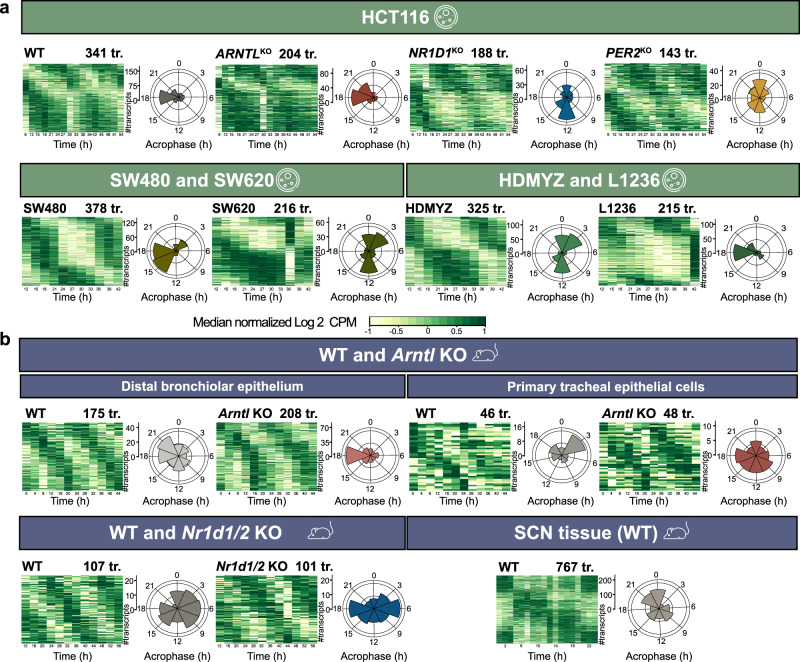
Circadian expression of splicing factors and spliceosome machinery related transcripts in mammalian RNA-seq datasets. Median normalized phase-sorted heatmaps (left) and acrophase bins (right) of SFs circadian transcripts (tr.) in (**a**) HCT116_WT and HCT116^KO^ cells (upper panel), SW480 and SW620 cell lines (lower left panel), HDMYZ and L1236 cell lines (lower right panel), **b** murine WT and *Arntl* KO from distal bronchiolar epithelium tissue (upper left panel) and from primary tracheal epithelial cells (upper right panel), murine WT and *Nr1d1/2* KO from epithelial cells (lower left panel), and murine SCN WT tissue (lower right panel).

To investigate possible alteration in SFs rhythmicity resulting from clock disruption, we examined the expression of SF transcripts and their rhythmic properties (Fig. 2). We compiled a total of 534 human SF genes from previously published studies^24–26,35,36^, Spliceosome database^37^, and SpliceAid 2 database^38^ (Supplementary file 1), and generated orthologous sets of human SFs for mouse (521 genes) using Ensembl Biomart^39^ (Supplementary file 2), and mapped the curated list of SFs against the circadian transcripts extracted from the RNA-seq datasets as depicted in the phase sorted heatmaps and acrophase bin plots (Fig. 2). We saw a decrease in the number of circadian expressed SF transcripts and also changes in their acrophase distribution in HCT116^KO^ compared to HCT116_WT cells (Fig. 2a; upper panel). HCT116_*PER2*^KO^ showed the lowest number of circadian rhythmic SF transcripts (143 transcripts; 104 genes) followed by HCT116_*NR1D1*^KO^ (188 transcripts; 135 genes), HCT116_*ARNTL*^KO^ (204 transcripts; 150 genes), and HCT116_WT (341 transcripts; 216 genes) datasets. Both HCT116_*NR1D1*^KO^ and HCT116_*PER2*^KO^ showed a shift in the SFs acrophase when compared with HCT116_WT cells.

To find a possible association between the decrease in the number of rhythmic SF transcripts and cancer progression, we analysed other CRC cell lines (SW480, SW620) and HL cell lines (HDMYZ, L1236). Indeed, we observed a decrease in the number of rhythmic SF transcripts in the metastasis-derived colon cancer (SW620: 216 transcripts; 153 genes) and in stage IV HL (L1236: 215 transcripts; 156 genes) as compared with the primary colon cancer (SW480: 378 transcripts; 212 genes) and stage III HL (HDMYZ: 325 transcripts; 201 genes) cell lines, respectively (Fig. 2a; lower panel). Also, the shift in acrophase distribution of circadian rhythmic SF transcripts was observed in both SW620 and L1236 cell lines compared with their controls. To further examine the impact of each KO on SFs rhythmicity, we examined rhythmic changes in SFs transcripts within murine RNA-seq datasets (Fig. 2b). In contrast, slightly higher number of rhythmic SF transcripts were observed in *Arntl* KO model of distal bronchiolar epithelium (208 transcripts; 151 genes) and primary tracheal epithelial cells (48 transcripts; 44 genes) compared with their WT (175 transcripts; 138 genes and 46 transcripts; 45 genes, respectively) (Fig. 2b; upper panel). Whereas, *Nr1d1/2* KO mouse model showed lower number of rhythmic SF transcripts (101 transcripts; 87 genes) compared with WT (107 transcripts; 87 genes) (Fig. 2b). SCN tissue, the master clock, showed the highest number of rhythmic SF transcripts (767 transcripts; 384 genes) in mouse WT datasets (Fig. 2b; lower panel, for the complete list of circadian transcripts and their acrophases see Supplementary Fig. 4). In addition, we also observed loss in 24 h rhythmic SFs due to *Cry1/2* deletion (Supplementary Fig. 5b; left panel) and *Per1/2* deletion (Supplementary Fig. 5b; right panel) vs. their controls in mouse datasets.

**Fig. 3:**
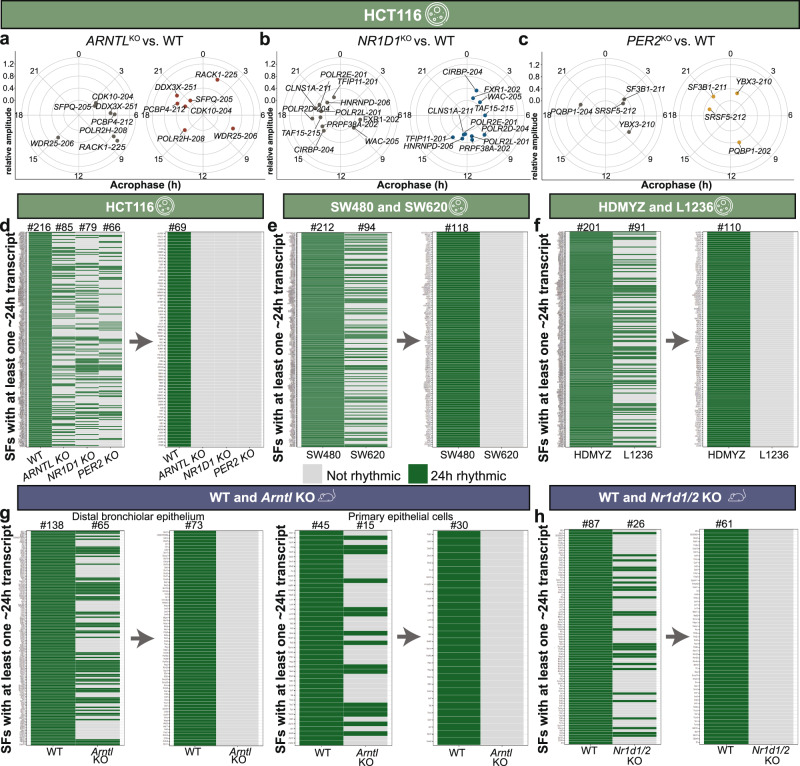
Clock disruption affected rhythmicity of splicing factors in human and murine datasets. **a**–**c** Circular plots depict the distribution of peak phases of overlapping differentially rhythmic SF transcripts between (**a**) HCT116_WT and HCT116_*ARNTL*^KO^, (**b**) HCT116_WT and HCT116_*NR1D1*^KO^, and (**c**) HCT116_WT and HCT116_*PER2*^KO^. **d**–**h** Categorial heatmaps represent the loss of rhythmic SFs in (**d**) HCT116^KO^ vs. WT, (**e**) SW620 vs. SW480, (**f**) L1236 vs. HDMYZ, (**g**) mouse *Arntl* KO vs. WT in distal bronchiolar epithelium (left) and primary epithelial cells (right), and (**h**) *Nr1d1/2* KO vs. WT. Green colour indicates SFs with at least one ~24 h transcript whereas grey colour indicates SFs with no rhythmic transcript. The numbers above the categorial heatmaps indicate the total number of SFs (genes) with rhythmic transcript(s).

The KOs resulted in a shift in the acrophase bin distribution of circadian rhythmic SF transcripts in particular for *NR1D1* and *PER2* KO. We further carried out pairwise comparisons of circadian SF transcripts between HCT116_WT and HCT116^KO^ to identify commonly circadian rhythmic SF transcripts, and to obtain differentially rhythmic SF transcripts (phase shift ≥3 h) in the HCT116^KO^ compared with HCT116_WT (Fig. 3a–c). A total of 25 circadian SFs transcripts (23 genes) were found rhythmic in HCT116_WT and HCT116_*ARNTL*^KO^, out of those, 7 transcripts showed differential rhythmicity (Fig. 3a). Besides alterations in the expression profile of SF transcripts, we observed phase shift in other transcripts, as well as loss of rhythmic oscillations in *PER1*, *PER2* and *DBP* gene with *ARNTL* disruption in HCT116 cells (Supplementary Fig. 6a).

Differential rhythmicity analysis between HCT116_WT and HCT116_*ARNTL*^KO^ cells resulted in a higher number of transcripts with phase difference greater than 9 h whereas for most differentially rhythmic splicing factor transcripts a phase difference between 6 and 9 h was observed (Supplementary Fig. 6b–f).

For HCT116_WT vs. HCT116_*NR1D1*^KO^, we found 33 circadian rhythmic transcripts (28 genes) and 11 circadian rhythmic transcripts to be differentially rhythmic (Fig. 3b). The comparison of HCT116_WT and HCT116_*PER2*^KO^ resulted in a total of 28 circadian expressed transcripts (26 genes) and out of those 4 transcripts showed differential rhythmicity (Fig. 3c). Only a single transcript from the gene survival of motor neuron 1 (*SMN1*), which plays a catalyst role in the assembly of small nuclear ribonucleoproteins (snRNPs) was found to be rhythmic in all HCT116 WT and KO datasets and it showed the same phase, but different amplitude within HCT116^KO^. Out of seven differentially rhythmic transcripts in HCT116_*ARNTL*^KO^ vs. WT, were SFs like *RACK1*, which is a component of the ribosomal subunit and Splicing factor proline and glutamine rich (*SFPQ*), essential at the early stages of spliceosome formation (Fig. 3a). For HCT116_*NR1D1*^KO^, differential rhythmic SFs transcripts were involved in pre-mRNA processing like *HNRNPD* and translational repressor like Cold Inducible RNA Binding Protein (*CIRBP*) (Fig. 3b). Whereas in HCT116_*PER2*^KO^, we found differentially rhythmic transcripts of SFs like *SRSF5*, which is a part of the spliceosome machinery (Fig. 3c).

To further understand the discrepancies in SFs rhythmicity, we extracted a list of SFs with at least one circadian transcript in HCT116_WT, SW480, HDMYZ, murine WT (from *Arntl* KO), murine WT (from *Nr1d1/2* KO), and mapped them with their corresponding knockout/altered condition (e.g. primary tumour- vs. metastasis-derived cells). We produced categorical heatmaps to visualize the loss of rhythmically expressed SFs in each dataset compared with controls (Fig. 3d–h). 216 SFs with at least one rhythmic transcript were observed in HCT116_WT, and less than 50% were rhythmic in HCT116^KO^ (Fig. 3d; left panel). Out of 216 SFs with at least one circadian transcript in HCT116_WT, we observed 85 SFs in HCT116_*ARNTL*^KO^, 79 SFs in HCT116_*NR1D1*^KO^, and 66 SFs in HCT116_*PER2*^KO^ with at least one circadian transcript. Among the three HCT116^KO^ cells, there were 69 common SFs with no rhythmic transcript (Fig. 3d; right panel). Similarly, the human CRC cell line (SW620), HL cell line (L1236), murine *Arntl* KO, and murine *Nr1d1/2* KO also showed a loss of rhythmic SFs compared with their controls (Fig. 3e–h). All three HCT116^KO^ cells showed loss of rhythmicity for *U2AF1*, an important RNA splicing mediator gene, which also lost rhythmicity in SW620 and L1236 cells, as compared to their corresponding lower grade cancer cell lines. We observed KO-specific loss of rhythmicity in different SF transcripts such as RNA Binding Motif Protein 5 (*RBM5*), Splicing factor 3a subunit 1 (*SF3A1*), and Serine and Arginine Rich Splicing Factor 3 (*SRSF3*), involved in the spliceosome machinery, uniquely in HCT116_*ARNTL*^KO^. While, HCT116_*NR1D1*^KO^ resulted in rhythmicity loss of SNW Domain Containing 1 (*SNW1*), another spliceosome component, and HCT116_*PER2*^KO^ resulted in the loss of rhythmic transcripts of *HNRNPC*, involved in pre-mRNA processing. Taken together, our results point to alterations in rhythmic properties of SFs in HCT116 cell lines, which were KO-specific and were also present in datasets derived from other cells and tissues, upon perturbation of the core-clock.

### Disruption of circadian clock elements influenced alternative splicing events across human and murine datasets

Aberrant AS events have been reported to be associated with different types of cancer^40^. We investigated the impact of clock disruption in our datasets on AS events with a potential impact in cancer onset and progression. We used SUPPA to calculate the proportion spliced-in (PSI) of seven basic modes of AS: (1) Alternative 3′ splice site (A3-event), (2) Alternative 5′ splice site (A5-event), (3) Alternative first exon (AF-event), (4) Alternative last exon (AL-event), (5) Mutually exclusive event (MX-event), (6) Retention intron event (RI-event), and (7) Skipping exon event (SE-event).

**Fig. 4:**
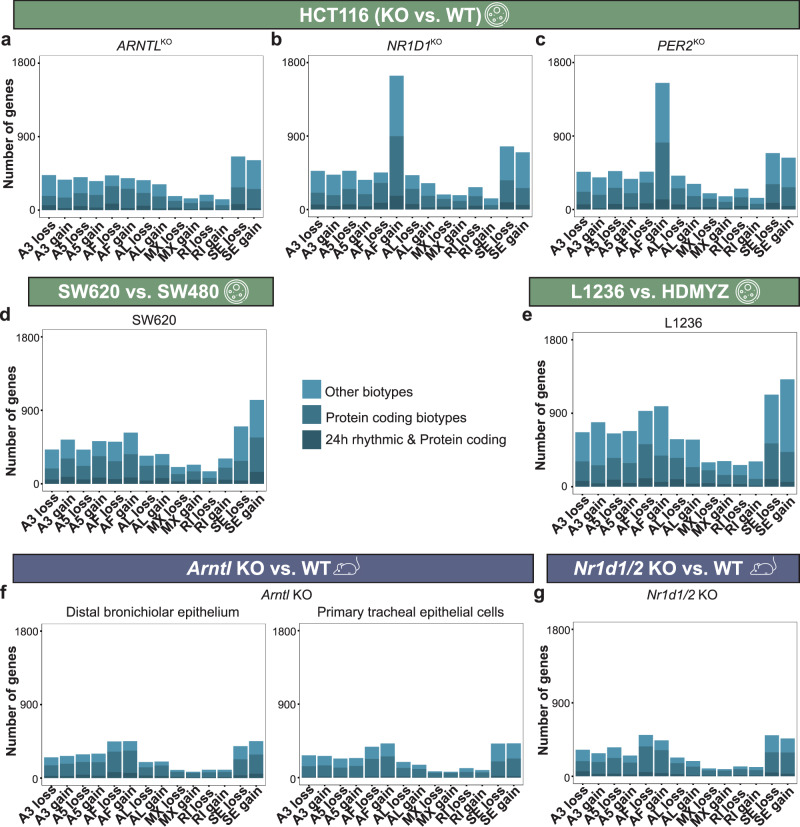
Local alternative splicing analysis revealed changes in the pattern of loss and gain of all seven splice modes in human and murine datasets. Bar plots depict the total number of genes (x-axis) with loss and gain of splicing (**a**–**c**) in the HCT116_*ARNTL*^KO^, HCT116_*NR1D1*^KO^, and HCT116_*PER2*^KO^ vs. HCT116_WT cells, (**d**) in the SW620 vs. SW480 cells, (**e**) in the L1236 vs. HDMYZ cells, (**f**) in *Arntl* KO from distal bronchiolar epithelium (left) and from primary tracheal epithelial cells (right) vs. their WT, and (**g**) in *Nr1d1/2* KO vs. WT. In each barplot, the number of genes containing transcripts with circadian expression and protein-coding biotype (dark blue), protein-coding biotype (blue), and different biotypes (light blue) is indicated.

We determined the overall number of genes showing significant AS events (0.1< PSI < 0.9), and found that most genes had a commonly predominant AS event (SE-event) in all human RNA-seq datasets (Supplementary Fig. 7). To further evaluate the changes in AS events caused by the KO of core-clock genes, we classified the set of genes that were alternatively spliced in the WT, but not in the KOs (following the PSI criteria), as “event loss” in its corresponding KO group. Similarly, uniquely identified sets of genes alternatively spliced in a KO cell line were labelled as “event gain” (see “Methods” for details). Bar plots were used to depict the total number of genes in the categories loss and gain for each type of splicing event (Fig. 4). We mapped the list of AS genes with the genes containing at least one circadian transcript and their biotypes. For better visualization, we separated the genes according to whether or not they contained transcripts with protein-coding biotype. All AS events in HCT116^KO^ cells except for the AF-event in HCT116_*NR1D1*^KO^ and HCT116_*PER2*^KO^ showed higher number of genes present in the loss of splicing events vs. gain of splicing events (Fig. 4a–c). However, a large increase in the number of genes with AF-gain was observed in both HCT116_*NR1D1*^KO^ (gain vs. loss: 75.8% vs. 24.4%) and HCT116_*PER2*^KO^ (gain vs. loss: 74.2% vs. 25.7%) (Fig. 4b–c). Interestingly, higher number of genes with gain in splicing events were observed in the metastasis CRC cell line (SW620) and in stage IV HL cell line (L1236) (Fig. 4d–e). We further investigated if the same genes were alternatively spliced through AF-event (gain) in both HCT116_*NR1D1*^KO^ and HCT116_*PER2*^KO^. Subsequently, we filtered for genes with at least one protein-coding transcript, carried out a functional enrichment analysis, and found biological processes such as protein acetylation enriched in common AF-gain candidates, positive regulation of cellular response to TGF-beta stimulus enriched in HCT116_*NR1D1*^KO^ unique candidates, and endothelial cell proliferation unique AF-event in HCT116_*PER2*^KO^ candidates (Supplementary Fig. 8).

A similar number of genes were observed in loss and gain of splicing events within the mouse *Arntl* KO dataset (Fig. 4f). In the case of *Nr1d1/2* KO model, a slightly higher number of genes were associated with a loss of splicing events than to a gain of splicing events (Fig. 4g). However, an increase in gain of AF-event as seen in HCT116_*NR1D1*^KO^ was not observed in the mouse *Nr1d1/2* KO. Taken together, we observed a distinct pattern in the number of genes being alternatively spliced in HCT116^KO^ vs. WT pointing to clock knockout specific alterations in alternative splicing.

Further, we carried out pairwise differential splicing analysis between KO and WT (Supplementary Fig. 9). Each differentially spliced candidate showed significantly (*p* < 0.05) higher change in transcript abundance in the KO than in the corresponding WT condition. Both HCT116_*NR1D1*^KO^ and HCT116_*PER2*^KO^ showed higher number of differentially spliced genes than in HCT116_*ARNTL*^KO^ cells (Supplementary Fig. 9a–c). Besides, the highest number of differentially spliced genes were observed in the SE-event for all cancer cell lines (Supplementary Fig. 9a–e). In murine KO models, a smaller number of differentially spliced candidates were observed (Supplementary Fig. 9f–g). We then searched for common differentially spliced candidates between the human and mouse KO datasets. Common differentially spliced candidates were observed only within SE-event. Sorting Nexin 3 (*SNX3*), which is involved in intracellular trafficking was found to be differentially spliced in both HCT116_*ARNTL*^KO^ cells and mouse *Arntl* KO from distal bronchiolar epithelium compared to their controls. *SNX3-201* and *SNX3-204* isoforms were present in HCT116_*ARNTL*^KO^. Among the common differentially spliced candidates between HCT116_*NR1D1*^KO^ and mouse *Nr1d1/2* KO, were Prolyl 4-Hydroxylase Subunit Alpha 1 (*P4HA1*), associated with oxidoreductase activity and Leucine Rich Repeat Containing 8 VRAC Subunit A (*LRRC8A*), which is an essential component of the volume-regulated anion channel, and plays a role in cell adhesion and cellular trafficking. *LRRC8A-202* and *LRRC8A-203* transcripts contributed to the observed differential splicing of *LRRC8A* and *P4HA1-202* and *P4HA1-203* transcripts contributed to differential splicing of *P4HA1*. While in the mouse *Nr1d1/2* KO, *Lrrc8a-201* and *Lrrc8a-202* isoforms contributed to the observed differential splicing of *Lrrc8a* and *P4ha1-202* transcript contributed to differential splicing of *P4ha1*. Taken together, our results showed a core-clock KO-specific impact on AS events within the datasets analysed.

### Core-clock KO in HCT116 cells resulted in the occurrence of differentially rhythmic transcript pairs associated with cancer hallmarks

**Fig. 5:**
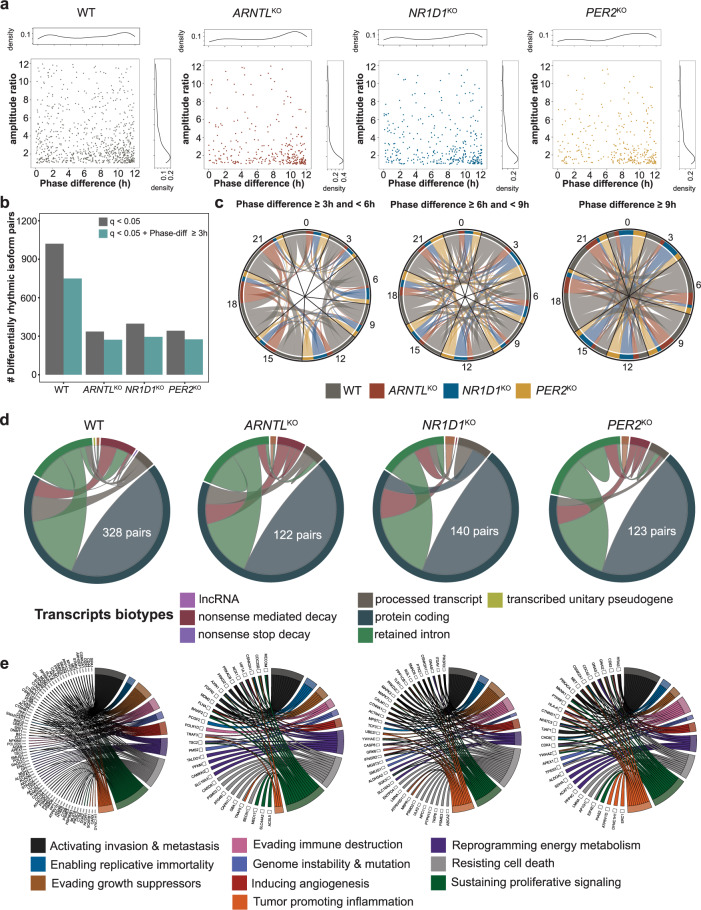
Phase-shifted spliced isoforms in HCT116 datasets are associated with hallmarks of cancer. Differential rhythmicity analysis between rhythmic transcripts within the same cell line was carried out using DODR. **a** The scatter plot depicts the distribution of differentially rhythmic transcript pairs according to their phase difference and amplitude ratio in HCT116. **b** Decline in differentially rhythmic isoform pairs observed in all HCT116^KO^. **c** The chord diagram represents the peak phases of transcript pairs across HCT116 under different phase shift cut-offs. **d** The chord diagram depicts the biotypes of phase shifted transcript pairs. **e** The circular plot shows the association of genes containing phase shifted transcript pairs with hallmarks of cancer.

Based on the differences in SFs rhythmicity and local AS events, as seen in HCT116^KO^, we would expect clock dependent changes in the rhythmicity of alternatively spliced transcripts. We used data from circadian transcripts to examine whether transcript isoforms from the same gene in each condition show phase-shifted rhythmic expression. For that, we extracted rhythmic and phase-shifted transcript pairs (Fig. 5). In short, we compared each rhythmic transcript with all other rhythmic transcripts of the same gene, in a pairwise manner, within the same cell line datasets. Differentially rhythmic transcript pairs (*q* < 0.05) were obtained for phase differences larger than 3 h. The scatter plot depicts the distribution of differentially rhythmic transcript pairs according to their phase difference and amplitude ratio (Fig. 5a). All HCT116^KO^ cells showed a decrease in the number of differentially rhythmic transcript pairs vs. HCT116_WT (Fig. 5b). 1021 transcript isoform pairs (592 unique genes) showed differential rhythmicity in HCT116_WT and out of those, 750 transcript pairs (469 unique genes) showed a phase shift >3 h. The lowest number of differentially rhythmic transcript pairs were seen in HCT116_*ARNTL*^KO^ (336 isoform pairs; 255 unique genes) and out of those, 272 transcript isoform pairs (220 unique genes) showed the pre-defined phase shift (Fig. 5b). 398 transcript isoform pairs (297 unique genes) were differentially rhythmic in HCT116_*NR1D1*^KO^ and out of those, 295 pairs (228 genes) had a phase shift larger than 3 h. HCT116_*PER2*^KO^ cells showed 342 differentially rhythmic isoform pairs (241 unique genes), and 275 pairs (196 unique genes had a phase shift larger than 3 h (Fig. 5b). To further explore the existence of different phase shifts within differentially rhythmic isoform pairs, we compared the peak phases of isoform pairs (Fig. 5c). A higher number of differentially rhythmic transcript pairs showed phase-difference ≥9 h (Fig. 5c; right panel) as compared with phase-difference ≥3 h and <6 h (Fig. 5c; left panel) and phase-difference ≥6 h and <9 h (Fig. 5c; middle panel) in all HCT116 datasets. To further understand the role of phase-shifted isoform pairs, we explored their biotypes (Fig. 5d). Transcript isoform pairs with protein-coding biotypes were observed as the largest pair in all HCT116 datasets. Among phase-shifted transcript pairs with protein-coding biotypes, we observed 328 pairs in HCT116_WT, 122 pairs in HCT116_*ARNTL*^KO^, 140 pairs in HCT116_*NR1D1*^KO^, and 123 pairs in HCT116_*PER2*^KO^. Also, transcript pairs with different biotypes such as nonsense-mediated decay or retained intron were phase-shifted in HCT116 datasets (Fig. 5d). For instance, three transcript pairs from Baculoviral IAP Repeat Containing (*BIRC5*), which promotes cell proliferation and prevents apoptosis, were differentially rhythmic only in HCT116_WT cells. Out of the three transcript pairs, two pairs consisted of protein-coding biotypes and one pair consisted of protein-coding and nonsense-mediated decay biotype. We observed two phase-shifted transcript pairs from Polycomb Group Ring Finger 2 (*PCGF2*), which controls cell proliferation specific to HCT116_*ARNTL*^KO^. Both the transcript pairs of *PCGF2* consisted of protein-coding biotypes. In HCT116_*NR1D1*^KO^, we also saw KO-specific phase-shifted transcript pairs with retained intron and nonsense-mediated decay biotypes from ATP Binding Cassette Subfamily A Member 2 (*ABCA2*), a lipid transporter. HCT116_*PER2*^KO^ showed two unique phase-shifted transcript pairs with protein-coding and retained intron biotypes from Chromodomain Helicase DNA Binding Protein 8 (*CHD8*), a transcription suppressor gene.

A functional enrichment analysis for genes with phase-shifted isoform pairs was carried out for HCT116 datasets (Supplementary Fig. 10). Genes with phase-shifted isoform pairs in HCT116_WT were enriched in processes like DNA damage response and G1/S cell cycle transition (Supplementary Fig. 10). Whereas, HCT116_*ARNTL*^KO^ phase-shifted candidates were uniquely enriched in processes like regulation of apoptotic signalling and EMT. HCT116_*NR1D1*^KO^ phase-shift candidates were uniquely enriched in processes like macrophage proliferation and intracellular cholesterol transport. HCT116_*PER2*^KO^ candidates were uniquely enriched in processes like regulation of intracellular transport and negative regulation of chromatin organization (Supplementary Fig. 10).

Subsequently, we mapped the genes with phase-shifted isoform pairs (phase-diff ≥3 h) with a cancer hallmark list of genes obtained from the cancer hallmark genes database^41^ (Fig. 5e). A total of 76 genes in HCT116_WT, 31 genes in HCT116_*ARNTL*^KO^, 38 genes in HCT116_*NR1D1*^KO^, and 30 genes in HCT116_*PER2*^KO^ with differentially rhythmic isoform pairs, were related to cancer hallmarks (Fig. 5e). Transcript pairs from *CSNK2A1* were observed in all HCT116 datasets. However, different phase-shifted transcript pairs were seen in HCT116_WT and HCT116^KO^. In HCT116_WT, we observed phase-shift transcript pairs from *SMAD2*, a cancer hallmark gene. Also, transcript pairs from *AKT2*, involved in all cancer hallmark processes except genome instability were phase-shifted in HCT116_WT. However, differentially rhythmic transcript pairs of both *SMAD2* and *AKT2* were not found in the KO cells. Phase-shifted transcripts pairs from *MET*, which controls several cancer hallmarks were seen in HCT116_WT and HCT116_*PER2*^KO^. In particular, *MET-202* and *MET-206* in HCT116_WT and *MET-203* and *MET-206* in HCT116_*PER2*^KO^ cells were differentially rhythmic. In HCT116_*ARNTL*^KO^, we observed a phase-shift in differentially rhythmic transcript pairs (from cancer hallmark genes) including *HIF1A* and *FGFR2* that were neither differentially rhythmic in HCT116_WT nor in other HCT116^KO^ (Fig. 5e). Similarly, we observed phase-shifted transcript pairs from cancer hallmarks genes like *HRAS* and *CD63* unique to HCT116_*PER2*^KO^. Moreover, we observed phase-shifted transcript pairs related to cancer hallmark genes like *CASP8* and Mitogen Activated Protein Kinase 3 (*MAPK3*) that were unique to HCT116_*NR1D1*^KO^ (Fig. 5e).

**Fig. 6:**
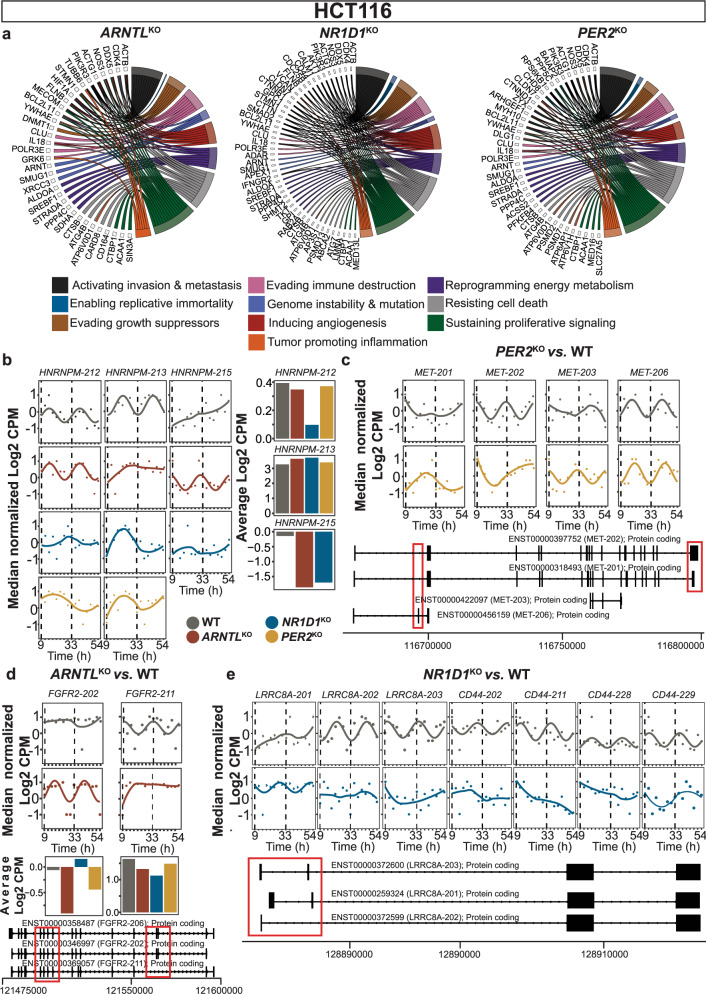
Clock disruption in HCT116 resulted in alterations in AS in genes involved in the hallmarks of cancer. Commonly spliced and uniquely spliced candidates in HCT116^KO^ were mapped to the cancer hallmarks gene list. **a** Circular plots depict the association between spliced candidates and different cancer hallmarks (**b**) *HNRNPM*, a spliceosome machinery component lost its circadian expression in HCT116^KO^ cells. HCT116^KO^ cells also showed discrepancies in the mean expression level of *HNRNPM* transcripts. Expression of uniquely alternatively spliced candidate transcripts in (**c**) HCT116_*PER2*^KO^, (**d**) HCT116_*ARNTL*^KO^, and (**e**) HCT116_*NR1D1*^KO^ were plotted. Genomic region plots of *MET*, *FGFR2* and *LRRC8A* transcripts represent differences in their exon composition (marked in red) compared to canonical forms. Circadian rhythmic transcripts were plotted using harmonic regression fit and arrhythmic transcripts were plotted using Loess fit in R.

The association of specific cancer hallmarks genes with differentially rhythmic isoform pairs due to different clock alterations in HCT116 motivated the subsequent analysis of alternatively spliced candidates and uniquely spliced candidates in all HCT116^KO^ cells. Therefore, we intersected the list of candidates that were differentially spliced, and found in the subsets of loss or gain of splicing event (rhythmic at least in HCT116_WT), and further shortlisted the candidates related to cancer hallmarks. For better visualization, we filtered cancer hallmarks genes for which at least two transcripts were rhythmic in HCT116_WT for loss of splicing event (Fig. 6a). Whereas, candidates with at least two rhythmic transcripts in KO were shortlisted for gain of splicing event. The complete list of candidates categorized based on cancer hallmarks is provided in Supplementary file 3.

*HNRNPM*, a component of spliceosome machinery and involved in pre-mRNA processing showed either a change in the rhythmic properties or a loss of rhythmicity in HCT116^KO^ vs. HCT116_WT cells (Fig. 6b). Moreover, we observed differences in the mean expression level of *HNRNPM* transcripts among HCT116 datasets. Both HCT116_*NR1D1*^KO^ and HCT116_*PER2*^KO^ resulted in the loss of rhythmicity of transcripts from *HNRNPM*. On the contrary, HCT116_*ARNTL*^KO^ resulted in the gain of one circadian *HNRNPM* transcript (*HNRNPM-215*) vs. HCT116_WT. When analysing knockout-specific spliced candidates, we observed cancer hallmarks genes like *MET* as uniquely spliced in HCT116_*PER2*^KO^ (Fig. 6c). The genomic region plots of *MET* transcripts (all protein-coding biotypes) represent the difference in coding exon composition compared to the canonical form of *MET* (*MET-202*). Moreover, the comparison of the coding exon sequence between the canonical form and other transcripts showed missing/skipping of exon 1- exon 21 (in *MET-206*), missing of exon 10 (in *MET-201*), and missing/skipping of all exons between 1 to 21 except exon 10,11 (in *MET-203*). The AS transcripts of cancer hallmarks genes like *FGFR2* showed differential rhythmicity unique to HCT116_*ARNTL*^KO^ (Fig. 6d). Specifically, HCT116_*ARNTL*^KO^ showed changes in the expression of two of the *FGFR2* transcripts (*FGFR2-202* and *FGFR2-211*; both protein-coding). The genomic plots for *FGFR2* transcripts show the loss of coding exon region in *FGFR2-202* and *FGFR2-211* vs. *FGFR2-206* (canonical form). The comparison of coding exons sequence between *FGFR2-206* and *FGFR2-202/-211* showed exclusion of exon 9 in *FGFR2-202* while exclusion of exon 8 in *FGFR2-211* (Fig. 6d). *LRRC8A* was differentially spliced in both HCT116_*NR1D1*^KO^ vs. HCT116_WT and mouse *Nr1d1/2* KO vs. WT. *LRRC8A-201* was not circadian in HCT116_WT, but it was circadian expressed in HCT116_*NR1D1*^KO^ (Fig. 6e). *CD44* was differentially spliced only in HCT116_*NR1D1*^KO^ vs. HCT116_WT, however, the resulting transcripts were rhythmic only in HCT116_WT. We also observed gain in rhythmic transcripts of cancer hallmark genes unique to specific HCT116^KO^ vs. HCT116_WT. For instance, rhythmic transcripts from Succinate Dehydrogenase Complex Flavoprotein Subunit A (*SDHA*), a complex of mitochondrial respiratory chain were unique to HCT116_*ARNTL*^KO^ vs. HCT116_WT. *SDHA-206* (retained intron) was rhythmic in HCT116_WT whereas *SDHA-202* (retained intron) and *SDHA-211* (protein-coding) were rhythmic only in HCT116_*ARNTL*^KO^. In case of HCT116_*NR1D1*^KO^, we observed a new rhythmic transcript of Diacylglycerol Kinase Zeta (*DGKZ*), a regulator of intracellular signalling. *DGKZ-215* and *DGKZ-218* (both with a retained intron event) were rhythmic in HCT116_WT whereas *DGKZ-201* (protein-coding) was rhythmic only in HCT116_*NR1D1*^KO^. Similarly, we saw that a new rhythmic transcript from Platelet Derived Growth Factor Subunit (*PDGFA*), which is essential for cell survival was unique to HCT116_*PER2*^KO^. *PDGFA-201* (protein-coding) was rhythmic in HCT116_WT and *PDGFA-203* (protein-coding) was only rhythmic in HCT116_*PER2*^KO^. Altogether, our findings showed that each of three core-clock knockouts resulted in aberrant alternative splicing of different cancer hallmarks related genes, pointing to a role for the circadian clock in the regulation of alternative splicing with potential consequences in tumorigenesis.

## Discussion

Pre-mRNA splicing contributes to generate diversity in the products of more than 95% human genes and leads to alternatively spliced transcripts that encode distinct proteins. This mechanism is often used to maintain cellular homeostasis and to regulate cell differentiation and development^42,43^. Previous studies have pointed to a regulation of AS via the circadian clock in cancer, suggesting a temporal pattern of AS, which affected the expression of alternatively spliced target genes in a time-dependent manner^24–27^. Interestingly, the distinct temporal AS pattern and the time-dependent expression of target genes were shown to correlate with the circadian phenotype of the investigated cancer cell lines. For example, SW480 colon cancer cells with a robust circadian clock showed twice as many genes with circadian alternative exons (59 genes) compared to the SW620 cell line (29 genes), the metastatic counterpart of SW480 from the same patient with a less robust circadian rhythm^24^. In addition, many of the affected alternatively spliced target genes with circadian AS patterns were involved in various key cancer pathways including *VEGFA* (involved in angiogenesis) and *CD44* (involved in EMT and metastasis), indicating a relevant role for temporal AS in mediating cancer progression.

In the current study, we evaluated the effect of circadian regulation of AS in the colon cancer cell line HCT116 with a robust circadian clock, as well as in core-clock knockout mutants of HCT116, and investigated the potential clock-AS interplay in cancer promoting properties. We also investigated our results in different colon cancer cell lines (SW480 and SW620), HL cells and healthy mouse tissues for a more general understanding of the interplay between alterations of the circadian clock and resulting AS events.

Our analysis showed that the alterations in core-clock components directly influenced the rhythmicity pattern of splicing factor transcripts, which subsequently impacted AS events. The SFs whose transcripts showed altered rhythmicity pattern in HCT116^KO^ included *RACK1, SFPQ* (altered in HCT116_*ARNTL*^KO^), *CIRBP*, *HNRNPD* (altered in HCT116_*NR1D1*^KO^), and *SRSF5* (altered in HCT116_*PER2*^KO^). Both *RACK1* and *SFPQ* regulate numerous cancer-related cellular processes.

*RACK1* is critical for cell proliferation and transcription^44^, while SFPQ is a multi-functional protein that regulates several processes such as RNA post-transcriptional activity^45^, splicing regulation^46^, and DNA repair^47^. Aberrant expression of SFPQ is associated with aetiology of colorectal cancer^48^. A previous study by Pellarin *et al* reported the reduction in platinum chemotherapy induced apoptosis due to *SFPQ* via alternative splicing of *CASP9* in human epithelial ovarian cancer samples^49^. Our analysis showed that *ARNTL* deletion in HCT116 cells resulted in a phase shift (>6 h) within *SFPQ* transcripts (Fig. 3, Supplementary Fig. 6), pointing towards its regulation via a core-clock component. Similarly, phase shift in *RACK1* transcript in HCT116_*ARNTL*^KO^ vs. WT suggests that its circadian variation is linked uniquely with *ARNTL*. A previous study using mouse fibroblasts reported the recruitment of RACK1 receptor in a circadian manner into the nuclear BMAL1 complex, and the overexpression of RACK1 suppressed CLOCK-BMAL1 transcriptional activity^50^. Similarly, BMAL1 was also shown to regulate the expression of *Sfpq* in rat pituitary cells^51^, thereby corroborating our findings.

In addition to the observed phase shift in SFs due to the KO of *ARNTL*, we also observed phase shifts among other transcripts. *CIRBP*, an RNA binding protein regulates several processes like cell proliferation and circadian gene expression^52^ while *HNRNPD* regulates mRNA stability of genes involved in the cell cycle^53^. The phase shift in rhythmic expression of *CIRBP* and *HNRNPD* transcripts seen in HCT116_*NR1D1*^KO^ suggests an effect of *NR1D1* depletion on the spliceosome machinery. Indeed, *Cirbp* was reported to regulate sleep and circadian clock via *Nr1d1* in mice^54^ and *Hnrnpd* was rhythmic in mice cells^26^. Yet, to our knowledge, no studies were found reporting *NR1D1*-mediated *HNRNPD* expression modulation. Likewise, the phase shift detected in *SRSF5* transcript in HCT116_*PER2*^KO^ pointed towards its regulation via *PER2*. Aberrant expression of *SRSF5* is associated with different cancer types and their severity^55^, however, no direct role of *PER2*-mediated *SRSF5* rhythmicity was reported before. Moreover, we observed loss of rhythmicity in several relevant components of the spliceosome complex including *U2AF1* and *MBNL2* (required for spliceosome binding to the pre-mRNA branch site and a modulator of AS, respectively) with deletion of clock components in all three HCT116^KO^ cells. Mutations in *U2AF1* contribute to cancer progression and have been reported in several different cancer types including CRC^56^, while *MBNL2* was reported as tumour suppressor in hepatocarcinogenesis^57^. Accordingly, the loss of *U2AF1* and *MBNL2* rhythmic transcripts, as seen in HCT116^KO^, point towards their possible contribution to metastasis formation. Indeed, our results also showed the loss of *U2AF1* and *MBNL2* rhythmicity in SW620 (metastasis CRC) and L1236 (stage IV HL).

Several studies reported that alterations in the spliceosome machinery lead to aberrant AS patterns^12,58–64^. In agreement with these data and our findings for HCT116 cells, we also found loss of rhythmicity in SFs genes in recently published RNA-seq datasets of murine *Per1/2* KO (GSE171975^65^) and murine *Cry1/2* KO (GSE135898^66^) (Supplementary Fig. 5). These results highlight the relevance of core-clock elements in the regulation of genes involved in splicing. Indeed, aberrant pre-mRNA splicing and alterations in splicing factors are known to act as oncogenic drivers and contribute to tumour progression^67^. Our results show the existence of alterations in rhythmic properties of SFs transcripts due to the KO of core-clock elements, which was reflected on the resulting AS transcripts. For instance, the pre-spliceosomal complex element *RBM5* regulates AS of *CASP9*, an apoptosis-related gene^4,68^ and showed altered rhythmic properties in HCT116_*ARNTL*^KO^ vs. HCT116_WT cells. *RBM5*, a tumour suppressor gene and splicing factor, improves the production of mRNAs by recognizing incorrect 3′splice sites of epidermal growth factor receptor (*EGFR*) pre-mRNA, thereby inhibiting the proliferation of tumour cells^69^. Previous studies in different cancer types suggested RBM5 as a potential target to prevent tumorigenesis^70,71^. In our study, we observed a regulation of *RBM5* expression via the circadian clock, which led to a loss of its rhythmicity due to *ARNTL* disruption.

The impact of circadian dysregulation in the splicing machinery was also seen through the loss/gain of AS events in all HCT116^KO^ cells. Among the different splicing events, exon skipping is the most common splicing event in human tissues^72^, as also observed in our study. The unique AF-event gain as seen in HCT116_*NR1D1*^KO^ and HCT116_*PER2*^KO^ indicates that the deletion of either *PER2* or *NR1D1* in HCT116 alters AS by preferring AF-event to the other six modes of AS.

By comparing our results to datasets from mouse models, we found *SNX3* (common between HCT116_*ARNTL*^KO^ and murine *Arntl* KO from distal bronchiolar epithelium tissue) and *P4HA1*, *LRRC8A* (common between HCT116_*NR1D1*^KO^ and murine *Nr1d1/2* KO) among the common differentially spliced candidates between HCT116^KO^ and murine KO models, however resulting in different sets of alternatively spliced transcripts. These differences suggest that the deletion of *ARNTL* or *NR1D1* in HCT116 is likely to have a different biological impact (protein product) compared to the same KOs in murine models. Differential splicing of *SNX3* in HCT116_*ARNTL*^KO^ and *P4HA1*, *LRRC8A* in HCT116_*NR1D1*^KO^ point towards splicing alterations, which are KO specific. *SNX3*, a unique mediator of WNT protein secretion, was reported to mediate EMT and metastasis in CRC cells including HCT116^73^. *P4HA1*, a catalytic enzyme, was reported to regulate cell proliferation in CRC cells via HIF1A and WNT signaling^6^. While, *LRRC8A*, a main regulatory subunit of VRAC (volume-regulated anion channel), was found to be upregulated in colon cancer patients and might contribute to metastasis^74^. However, the direct correlation between these genes and core-clock components remains unexplored.

Following the temporal regulation of different AS events in HCT116 datasets, our analysis revealed several transcript pairs that showed differential rhythmicity. Moreover, we also showed the occurrence of new KO-specific alterations in transcripts rhythmicity vs. WT. In HCT116_WT, phase-shifted isoforms were found to be enriched in DNA damage response and G1/S cell cycle transition. This suggests a temporal regulation of DNA repair and cell cycle pathways that might be regulated by circadian AS events. Indeed, a previous study reported splicing as an emerging pathway contributing to DNA damage response^75^. In the KO cells, we observed different enriched processes among phase-shifted isoforms such as apoptotic signalling and EMT (HCT116_*ARNTL*^KO^), macrophage proliferation and intracellular cholesterol transport (HCT116_*NR1D1*^KO^), intracellular transport and chromatin organization (HCT116_*PER2*^KO^). These results highlight the individual impact of each of the core-clock KOs in cellular functioning. For instance, we identified phase shifted transcript pairs from different cancer hallmark genes such as *BIRC5* in HCT116_WT, *PCGF2* in HCT116_*ARNTL*^KO^, *ABCA2* in HCT116_*NR1D1*^KO^, and *CHD8* in HCT116_*PER2*^KO^. *BIRC5*, an immune-related gene was found to be highly expressed in different tumour types and promotes cell proliferation^76^. Moreover, *Birc5* was reported rhythmic in colon mucosa cells and its silencing resulted in an increased sensitivity of HCT116 cells to CDK inhibitors^77^. *PCGF2*, a ring finger protein, was reported to negatively regulate granulocyte differentiation in human HL-60 cells^78^. While, increased expression of *ABCA2*, a member of ATP transporters, was reported in different cancer types^79^. A previous report on *CHD8*, a negative regulator of Wnt signalling, showed that loss of its expression might be an indicator of aggressiveness in gastric cancer^80^. However, the interdependence of *PCGF2*, *ABCA2*, and *CHD8* on specific core-clock elements was not reported. We also observed phase-shifted isoforms of some gene such as *CSNK2A1* in all HCT116 datasets, however, different transcript pairs were phase-shifted in each KO, pointing to a KO-specific effect. Moreover, we saw the loss of differentially transcript pairs of cancer hallmark genes including *SMAD2* and *AKT1* in all HCT116^KO^. We also observed differential rhythmicity in certain cancer hallmark genes, that were not circadian in HCT116_WT. For instance, *HIF1A* and *FGFR2* showed differentially rhythmic transcript pairs unique to HCT116_*ARNTL*^KO^. *HIF1A* is the main element of the HIF1-pathway, which plays a crucial role in adaptive responses of tumour cells to hypoxia and promotes tumour progression and metastasis via oncogenic growth factors such as TGFß (transforming growth factor beta) and EGF (epidermal growth factor)^81^. *FGFR2* is an EMT-associated gene which encoded for two isoforms, IIIb and IIIc, characteristic to epithelial and to mesenchymal cells, respectively^82^. The discrepant expression pattern, as well as rhythmic properties of these two *FGFR2* spliced variants in HCT116_*ARNTL*^KO^ vs WT suggests that *ARNTL* deletion in HCT116 might promote metastasis via EMT.

Similarly, we observed differentially rhythmic transcript pairs of *HRAS* (proto-oncogene and member of the RAS-pathway involved in cell proliferation) and *CD63* unique to HCT116_*PER2*^KO^. *CD63* is a member of the tetraspanin family involved in cell differentiation and migration, which was found to be a prognostic marker in colorectal and esophageal cancer^83,84^. In HCT116_*NR1D1*^KO^, we observed unique differentially rhythmic transcript pairs of *CASP8* and *MAPK3*. *CASP8* is an apoptosis-related cysteine peptidase and an essential part of the death-inducing signalling complex (DISC)^85^. In cancer, it promotes proliferation and angiogenesis through the activation of NF-kB in glioblastoma^86^ and breast cancer^87^. *MAPK3* encodes for a member of the MAP kinase family (aka extracellular signal-regulated kinases (ERK)) and regulates various cellular processes such as proliferation, differentiation, and cell cycle progression in response to a variety of extracellular signals. These results point towards a cross-talk between aberrant AS events and cancer hallmarks related to each of the HCT116^KO^.

We also observed cancer hallmark genes like *MET*, whose different transcript pairs were phase shifted in HCT116_WT and HCT116_*PER2*^KO^. The *MET* oncogene encodes for a receptor tyrosine kinase with pleiotropic functions in initiating and sustaining neoplastic transformation, as well as in cancer cell survival and tumour dissemination^88^. MET is known to undergo AS and its AS isoform (lacking exon 14) is known to inhibit HGF-induced tyrosine phosphorylation of Met, as well as cell proliferation and migration in skeletal muscle myoblasts^89^.

We further compared our list of AS spliced candidates to a list of known cancer hallmark genes^41^. Of these, *ACTB* was differentially spliced in all HCT116^KO^ vs WT, however rhythmicity of *ACTB* transcripts were lost in all three KOs. This suggests that the loss in circadian profile of *ACTB* transcripts may contribute towards metastasis due to clock alterations. A previous study reported that the abnormal expression and polymerization of *ACTB* contributes to invasiveness and metastasis of different cancer types^90^. Besides different cancer hallmarks related to AS candidates, we also observed discrepant expression of *HNRNPM* SF transcripts in all three HCT116^KO^ vs. WT. Among different KO-specific AS candidates, *FGFR2* (in HCT116_*ARNTL*^KO^), *MET* (in HCT116_*PER2*^KO^), *CD44* (in HCT116_*NR1D1*^KO^) lost/gained rhythmicity in the KOs. In HCT116_*ARNTL*^KO^, *FGFR2* IIIb isoform (exon 9 exclusion and exon 8 inclusion; *FGFR2-202*) was circadian while in HCT116_WT *FGFR2* IIIc (exon 8 exclusion and exon 9 inclusion; *FGFR2-211*) was circadian. These discrepancies were better seen when we analysed the average expression of both isoforms in WT and HCT116_*ARNTL*^KO^ cells. The lower average expression of *FGFR2* IIIb in HCT116_*ARNTL*^KO^ while higher expression of *FGFR2* IIIc point to possible alterations in EMT signalling. Matsuda Y *et al*. reported increased FGFR2 IIIc expression among colorectal carcinomas samples and a human anti-FGFR2 IIIc monoclonal antibody was reported to inhibit growth in colorectal carcinoma cells^91^. These findings suggest that FGFR2 IIIc could be a promising therapeutic target for colorectal carcinoma.

*CD44* encodes for a family of cell adhesion molecules involved in homotypic and heterotypic interactions with extracellular matrix components, as well as EMT^92^. It promotes proliferation and invasiveness of cells via recruiting ERM proteins (Ezrin, Radixin und Moesin) under certain conditions. However, CD44 can also act as a tumour suppressor, for example when cells reach confluent growth conditions, and thus inhibit cell growth. The prognostic value of the CD44 variant 6 (CD44v6) in CRC was debated for years due to contradictory results^93–95^. The function of CD44 in a cell is determined by the CD44 isoform pattern expressed^15^. Several studies showed the relevance of CD44v6 as an independent negative prognostic factor and a promising therapeutic target in CRC^96,97^. In our study, we showed that *NR1D1* disruption in HCT116 cells results in aberrant alternative splicing of *CD44*, which points to a clock specific regulation of *CD44* splicing, and could potentially play a role in CD44-targeted therapy regimens. Interestingly, several CD44-targeted drugs have been approved for clinical trials, which highlights the importance of timing treatment in cancer therapy targeting CD44 (reviewed in^98^). Our results suggest that the perturbations in core-clock elements result in different AS outcomes for this gene.

Furthermore, certain genes showed a gain of new rhythmic transcripts in the KO vs. WT. These included *SDHA* (HCT116_*ARNTL*^KO^ vs. WT), *DGKZ* (HCT116_*NR1D1*^KO^ vs. WT), and *PDGFA* (HCT116_*PER2*^KO^ vs. WT). *SDHA* is responsible for transferring electrons from succinate to ubiquinone (coenzyme Q), and acts as a tumour suppressor and inhibitor of angiogenesis in paraganglioma^99^. *DGKZ* attenuates protein kinase C activity by regulating diacylglycerol levels in intracellular signalling cascade and signal transduction. It has proven to be associated with various signalling pathways, including ERK and MYC and acts as a potential oncogene in osteosarcoma^100^. *PDGFA* is a member of the PDGF as well as VEGF signalling. Paracrine PDGF signalling is commonly observed in epithelial cancers, where it triggers stromal recruitment and may be involved in EMT affecting tumour growth, angiogenesis, invasion, and metastasis. Overexpression of *PDGF* signalling was shown to drive tumour cell growth and to promote tumorigenesis in colorectal cancer, breast cancer, lung cancer and sarcomas (reviewed in ref. ^101^). The gained rhythmicity of a coding transcript of *SDHA* due to *ARNTL* deletion, *DGKZ* due to *NR1D1* deletion, and *PDGFA* due to *PER2* deletion in HCT116 may lead to aberrant protein functions resulting from clock-KO-specific splicing. However, the biological role of these transcripts remains to be elucidated.

The relevance of timing treatment for increasing tolerability and efficacy while minimizing toxic side-effects has been largely demonstrated for numerous anticancer agents in experimental models, as well as in clinical settings including clinical studies in patients with metastatic colorectal cancer^102–104^. Aberrant alternative splicing events and circadian clock disruption are reported in several different cancer types, adding an additional complexity to the genetic landscape of cancer^67,105^. The results of our study highlight the importance of timed AS, especially in genes regulating cancer hallmarks that are shown to be suitable drug targets. Our data point to the regulation of AS patterns in cancer via the circadian clock. It would be important to further explore these findings and to consider drug timing, at least for drugs targeting such genes, in future clinical studies.

Altogether, the results of our study suggest an interplay between circadian clock elements and AS in cancer, with distinct and unique roles for the core-clock genes *ARNTL*, *PER2* and *NR1D1* in regulating SF rhythmicity and AS events in cancer hallmark genes, with relevance in cancer onset and progression.

## Methods

### Cell culture

Human colorectal carcinoma cell line HCT116 (ATCC® CCL-247™, Gaithersburg, MD, USA) was cultured in Dulbecco’s Modified Eagle Medium (Gibco, Thermo Fisher Scientific, Waltham, MA, USA) supplemented with 10% Fetal Bovine Serum (Gibco, Thermo Fisher Scientific, Waltham, MA, USA) and 1% Penicillin–Streptomycin (Gibco, Thermo Fisher Scientific, Waltham, MA, USA) in an incubator with 5% CO_2_ at 37 °C. LUNA™ Automated Cell Counter (Logos Biosystems, Anyang, South Korea) was used for cell counting and morphology analysis. Cell lines were tested for mycoplasma by using the Mycoplasma check service of Eurofins Genomics (Eurofins Genomics, Ebersberg, Germany).

### CRISPR-Cas9 Knockout generation

A CRISPR-Cas9 mediated approach was applied to generate the core-clock knockout cell lines in HCT116. Briefly, HCT116_WT cells were seeded in 6-well plates at a density of 4 × 10^5^ cells/well and transfected with CRISPR-Cas9 plasmids containing GFP marker and guided RNAs targeting multiple exons of *ARNTL*, *PER2* or *NR1D1* genes, respectively. Cell transfection was performed using FuGENE HD Transfection Reagent (Promega Corporation, Fitchburg, WI, USA) according to the manufacturer’s instructions. 48 h post-transfection, CRISPR/Cas9 GFP-positive cells were single-cell sorted using an S3e cell sorter (Bio-Rad laboratories, Hercules, CA, USA) into 96-well plates. Colonies were expanded for subsequent testing and successful knockout colonies were used for the time-course RNA-seq experiment.

For each knockout condition, several single clones were investigated on RNA gene expression level to characterize and confirm the knockout. All KO cell lines displayed significantly reduced target gene expression compared to WT (Supplementary Fig. 1d). The off-target activity was investigated using Off-Spotter^106^ and the Welcome Trust Sanger Institute Genome Editing database (WGE)^107^ to search for the most likely potential off-target sites using gRNA sequences. Off-target sites with up to three mismatches within protein-coding regions were Sanger-sequenced and compared to WT. All investigated potential off-target sites in KO cells showed 100% sequence similarity to the WT, indicating absent off-target modifications. For regions where it was not possible to design specific primers amplifying the off-target site, we compared band sizes on gel electrophoresis.

### Sample preparation and RNA extraction

HCT116 cells were seeded in triplicates in 12-well plates with a density of 2 × 10^5^ cells per well. On the next days, cells were synchronized by changing the media. Sampling was started 9 h after synchronization and samples were taken every 3 h for a time-series of 45 h. To prepare the cells for RNA extraction, media was discarded and cells were washed with phosphate buffer saline (PBS) and lysed using RLT Plus buffer (Qiagen, Hilden, Germany) directly on the plate. A total of 64 samples were obtained from the HCT116 datasets (WT and three KOs).

Total RNA was isolated using the RNeasy Plus Mini kit (Qiagen, Hilden, Germany) according to the manufacturer’s guidelines. Genomic DNA was digested from the cells using gDNA eliminator columns provided with the kit (Qiagen, Hilden, Germany). RNA was eluted in 30 µL RNase-free water. RNA concentration was measured using a Nanodrop 1000 (Thermo Fisher Scientific, Waltham, MA, USA). RNA was then stored at −80 °C until further use.

### Data generation

High-quality RNA was used to generate mRNA libraries and prepared using the TruSeq Stranded mRNA Sample Preparation Kit (Illumina, San Diego, CA, USA) according to the sample protocol guidelines and sequenced on an Illumina NextSeq 500 platform to an average depth of 100 M 75-bp paired-end reads at the European Molecular Biology Laboratory (EMBL) GeneCore Facility (Heidelberg, Germany).

### Acquisition of additional RNA-seq datasets

Using a strict criterion of “paired-end RNA-seq”, “less than or equal to 4 h interval”, “mammalian tissue”, “circadian rhythm”, we obtained a total of 134 samples from six different datasets. Published paired-end RNA-seq for *Per1/2* was not available, therefore we included for this dataset recently published single-end *Per1/2* KO (24 samples). Out of seven different datasets, two datasets were retrieved from human model system and five datasets were retrieved from the mouse model system.

Based on human model system, RNA-seq datasets were collected derived from: (1) human CRC cell lines (SW480: primary tumour, SW620: metastatic tumour; Accession number: E-MTAB-7779) from the same patient, and (2) Hodgkin’s lymphoma (HL) cell lines of cancer progression stages (HD-MY-Z: stage IIIB, L-1236: stage IV; Accession number: GSE16206) from different patients. CRC cells (SW480, SW620) and HL cells (HDMYZ, L1236), which represent different progression grades of CRC and HL, respectively, display various clock phenotypes (Supplementary Fig. 2e). These datasets were chosen to find a possible correlation between clock disruption and cancer progression stage.

Based on mouse model system, we retrieved RNA-seq datasets derived from (1) mouse embryonic stem cells in WT condition and the *Nr1d1/2* KO (Accession number: GSE125696) generated using CRISPR/Cas9, (2) laser micro-dissected SCN tissue of male C3H/HeH wild type mice (age: 10–12 weeks; Accession number: GSE72095), (3) distal bronchiolar epithelium tissue isolated using laser capture microdissection from WT mice and *Arntl* KO mice bearing the targeted deletion in mouse club cells (age: 10–20 weeks; Accession number: E-MTAB-6384), and primary tracheal epithelial cells isolated from WT mice and *Arntl* global KO (age: 10–20 weeks; Accession number: E-MTAB-6384). In addition, we retrieved raw gene-level count data from two murine core-clock KO RNA-seq datasets from NCBI-GEO, liver tissue from WT and *Per1/2* KO mice (age: 3 months old, Accession number: GSE171975; single-end RNA-seq), and liver tissue from WT and *Cry1/2* KO mice under normal feeding (age: 9–14 weeks old, Accession number: GSE135898). All datasets listed in this section were downloaded from NCBI-GEO or ArrayExpress.

### Synchronization and sampling protocols of used RNA-seq datasets

E-MTAB-6384 (mouse *Arntl*-KO lung samples pulmonary airway epithelial cells): For circadian sampling, mice were maintained in constant darkness and samples collected 1 cycle after transfer to darkness (1 day later) at circadian time (CT), which by convention anchors expected time of lights off and activity onset to CT12. Samples were taken every 4 h for 48 h in constant dark conditions^108^.

GSE72095 (mouse SCN samples): After 7 days of acclimatisation, mice were singly housed for 7 days prior to tissue harvesting. At each sampling time-point, pooled dissected tissue from five adult male mice were used. Samples were taken for a total of six time-points over a 12:12 LD cycle at 4 h intervals (ZT2, 6, 10, 14, 18 and 22), where ZT0 denotes the time of lights on^109^.

GSE125696 (*Nr1d1/2* KO mouse ESCs): On differentiation day 28, cells were treated with 100 nM dexamethasone and frozen at the indicated time points. Time course RNA-Seq was performed using RNA samples at 4 h intervals over 2 days^110^.

Synchronization of HCT116 RNA-Seq samples: HCT116 cells were synchronized via medium exchange (which serves as a strong entrainment signal aka Zeitgeber in these cells). Previous reports on the efficacy of synchronization agents (e.g. dexamethasone, forskolin, serum shock, medium change) have shown, that medium exchange is as effective as other mentioned agents in synchronizing the circadian clock, as shown for different CRC cell lines via bioluminescence measurements of *ARNTL-*promoter activity^111^.

### Curation of splicing factor list

In total 534 human spliceosome and splicing-related genes were compiled from the literature^24,35,36^, as well as from online databases (Spliceosome database^37^ and SpliceAid 2^38^). A list of human splicing factors was mapped using Ensembl Biomart^39^ to obtain their orthologous sets for mouse. A total of 521 SFs were obtained for mouse. The complete list of splicing factors is provided in Supplementary files 1–2.

### RNA-sequencing data processing

All RNA-seq datasets were processed and analysed according to the following protocol unless otherwise stated. Quality assessment of raw reads was carried out using fastqcr (version 0.1.2) R package which easily parse, aggregate, and analyse FastQC^112^ for large number of samples. Based on FastQC reports, over-represented sequences and residual adapter sequences were trimmed from raw reads using Trimmomatic (version 0.39^113^) using default settings. After trimming, only paired-end reads were used for further downstream analysis. Paired-end reads from human datasets were aligned to human genome (Homo sapiens GRCh38, Ensembl release 92) and from mouse datasets were aligned to mouse genome (Mus musculus GRCm38, Ensembl release 102) using STAR aligner (version 2.6.0^114^). Based on STAR transcriptome alignment, transcript-level abundances were quantified in transcripts per million (TPM) using Salmon (version 0.10.2^115^). Afterwards, tximport R package^116^ was used to import transcript level abundance, estimate counts and transcript lengths. Tximport summarizes the quantification results into matrices for downstream analyses. Tximport can be used to obtain both transcript level (txOut = TRUE) and gene-level (txOut = FALSE) summarization. Here, we used transcript-level counts for detailed alternative splicing analysis. Nevertheless, gene-level summarization was also used for its comparison with transcript-level analysis. Trimmed mean of M-values (TMM) method of averaging was used as the normalization factor to scale up the raw library size using edge R package (version 4.0^117^). Counts per million (CPM) function was used as a descriptive measure of transcript expression. All transcript feature with at least 0.5 CPM on average over all time points (specific to datasets) were retained and renormalized using selected features. The complete pipeline for the analysis is depicted in Fig. 1.

Raw unlogged gene-level count data from *Per1/2* KO (GSE171975; single-end RNA-seq) and *Cry1/2* KO (GSE135898; paired-end RNA-seq) were downloaded from NCBI-GEO and used for rhythmicity analysis.

### Rhythmicity analysis

Unlogged CPM values were used to detect rhythmic signals from time-series datasets. 24 h rhythmicity was evaluated through a non-parametric method using RAIN R package (version 1.24.0^118^). Rhythmic gene/transcript-sets were obtained using a cut-off of q < 0.05. The acrophase and relative amplitude were estimated for rhythmic gene-/transcript-sets using Cosinor^119^ within Discorhythm R package (version 1.6.0^120^). The rhythmic gene/transcript-sets were further filtered using an additional cut-off of relative amplitude (rAMP) ≥ 0.1.

### Alternative splicing analysis using SUPPA

To calculate the local alternative splicing events based on the expression of transcripts in each dataset, we used SUPPA2^121^. SUPPA2 is helpful in studying splicing at the local alternative splicing level or at the transcript isoform level. We analyzed seven alternative splicing events types; (1) Alternative 3′ splice site (A3-event) where 3′ site acts as an acceptor, (2) Alternative 5′ splice site (A5-event) where 5′ site acts as a donor, (3) Alternative first exon (AF-event) where the first exon is retained after splicing, (4) Alternative last exon (AL-event) where the last exon is retained after splicing, (5) Mutually exclusive event (MX-event) where one of two exons is retained, (6) Retention intron event (RI-event) where the intron is confined within mRNA, and (7) Skipping exon event (SE-event) where an exon may be spliced out or retained.

For each gene in a given tissue or condition, the average percent spliced-in (PSI) value was calculated. Alternative splicing of a gene in a particular dataset is considered only if it fulfils the criteria of PSI < 0.9 and > 0.1. Using the PSI cut-off, we detected specific local events which only happened either in the WT or in the KO datasets. To classify these events, we grouped the local events as gain or loss in the KO with respect to its control. If a KO contained a gene which is spliced by a particular local event and the WT did not show the gene in that particular event, the event would match to gain in the KO. Similarly, if the control group contained a gene which is alternatively spliced by a particular local event and the corresponding KO did not show the gene in that particular event, the event would correspond to loss in the KO.

Besides, we also used SUPPA2 to carry out the differential splicing analysis where a pairwise comparison between the KO and WT was made. The differentially spliced candidates were obtained using p-val < 0.05.

### Differential rhythmicity analysis

The detection of differentially rhythmic transcripts isoform within each dataset was carried out using DODR R package (version 0.99.2^122^). All 24 h rhythmic transcript sets were used for this analysis. 24 h differentially rhythmic transcript isoform pairs per single tissue/condition type were obtained using FDR corrected p-value cut-off meta. *p* < 0.05. To further examine the transcript isoform with more prominent changes in HCT116, phase difference cut-off ≥3 h was applied.

### Acquisition of list of cancer hallmark genes

To understand the direct influence of clock alterations on cancer hallmarks, we retrieved the complete gene list categorized according to processes from Cancer Hallmark Genes database^41^.

The CHG database^41^ was established by systematically integrating manually curated hallmarks genes from the confirmed literature and KEGG pathways^123^. In parallel, based on pre-defined keywords related to hallmarks, a text mining software Lucene (https://lucene.apache.org/) was used to identify hallmark specific pathways in the literature and in the KEGG database. To establish the CHG database Zhang et al. used: (1) 301 KEGG pathways for Lucene search and the extraction of pathways genes, (2) Gene variant data (7075 mutation samples, 6177 methylation samples, 9445 copy number variation samples) from TCGA (The Cancer Genome Atlas https://www.cancer.gov/tcga) to calculate the frequency of gene variation, (3) Human protein-protein interaction data from HPRD (Human Protein Reference Database^124^), STRING^125^, BioGRID^126^, HTRIdb^127^ to integrate a gene interaction network. CHG provides the corresponding gene sets for ten cancer hallmarks: (1) Activating invasion and metastasis (1,149 genes), (2) Enabling replicative immortality (308 genes), (3) Evading growth suppressors (709 genes), (4) Evading immune destruction (609 genes), (5) Genome instability and mutation (231 genes), (6) Inducing angiogenesis (507 genes), (7) Reprogramming energy metabolism (461 genes), (8) Resisting cell death (1192 genes), (9) Sustaining proliferative signaling (1314 genes), and (10) Tumor promoting inflammation (648 genes).

### Functional enrichment analysis

Gene ontology analysis for selected gene sets was carried out using an R based package ClusterProfiler (version 4.0^128^). Enriched biological processes were obtained for each of the gene sets. Dot plots were used to visualize the top 10 enriched biological processes in the case of differentially rhythmic isoform pairs. The enriched biological processes for the candidate cancer hallmarks markers were manually curated after removing the redundant biological processes. The visualization of cancer hallmarks connected with selected candidate markers was carried out using an R based package GO plot (version 1.0.2^129^).

## Supplementary information

Supplemental Material

Supplementary_file_1

Supplementary_file_2

Supplementary_file_3

## Data Availability

The datasets analysed during the current study are available as follows: recently generated RNA-sequencing data for HCT116 WT cells and KOs in this study have been deposited in the ArrayExpress repository (Accession number: E-MTAB-9701). Other datasets used in this manuscript are available as follows: SW480 and SW620 cell lines, ArrayExpress, E-MTAB-7779; HD-MY-Z and L-1236 cell lines, Gene Expression Omnibus (GEO) GSE16206; mouse *Arntl*-KO lung samples Pulmonary airway epithelial cells, ArrayExpress, E-MTAB-6384; mouse SCN samples, GEO, GSE72095; *Nr1d1/2* KO mouse ESCs, GEO, GSE125696; murine *Per1/2* KO liver samples, GEO, GSE171975; murine *Cry1/2* KO liver samples, GEO, GSE135898.

## Code availability

All programs, packages and functions used in this manuscript are freely available and are referred to in the Method section.
