# Supplementary material for: Transcriptome analysis of clock disrupted cancer cells reveals differential alternative splicing of cancer hallmarks genes
Source: NPJ Syst Biol Appl. 2022 May 12;8:17. doi: 10.1038/s41540-022-00225-w (PMC9098426; doi:10.1038/s41540-022-00225-w)
Supplement: Supplementary file 1 — Supplemental Material [file 41540_2022_225_MOESM1_ESM.pdf]

# Supporting Information

## Transcriptome analyses of clock disrupted cancer cells reveals differential alternative splicing of cancer hallmarks genes


Deeksha Malhan[1,2,3], Alireza Basti[1,2,3], Angela Relógio[1,2,3*]

[1] Institute for Theoretical Biology (ITB), Charité – Universitätsmedizin Berlin, corporate member of Freie Universität Berlin, Humboldt – Universität zu Berlin, and Berlin Institute of Health, Berlin 10117, Germany

[2] Molecular Cancer Research Center (MKFZ), Medical Department of Hematology, Oncology, and Tumor Immunology, Charité – Universitätsmedizin Berlin, corporate member of Freie Universität Berlin Humboldt – Universität zu Berlin, and Berlin Institute of Health, Berlin 10117, Germany

[3] Institute for Systems Medicine, Faculty of Human Medicine, MSH Medical School Hamburg, Hamburg 20457, Germany

# Supplementary Figures

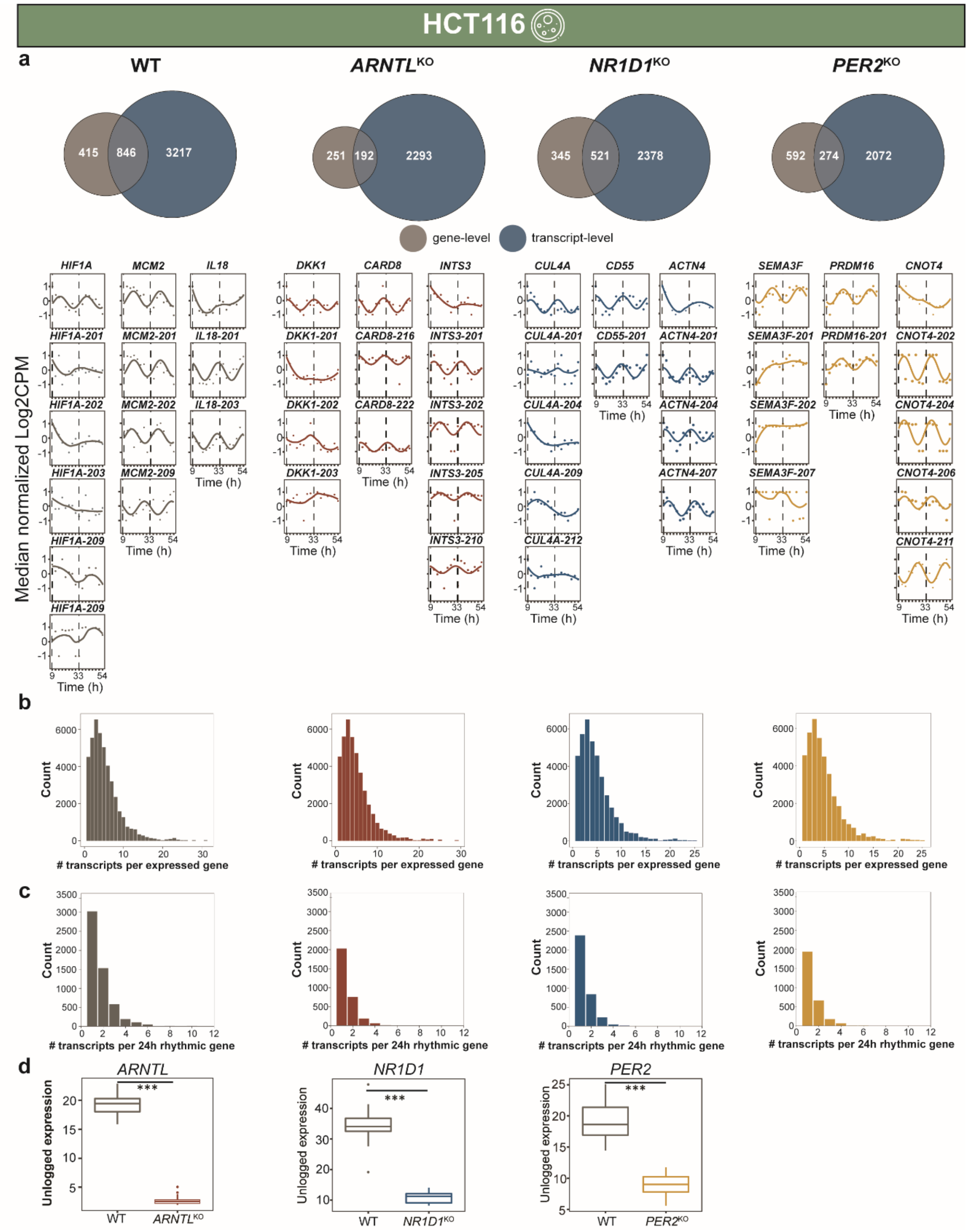


**Supplementary Figure 1: Comparison of 24h rhythmic sets identified at gene-level vs. transcript-level and characterization of core-clock KO expression in HCT116. a** Venn diagram (upper panel) depicts the overlap between the 24h rhythmic sets identified at the gene-level (grey) and at the transcript-level (dark blue) in HCT116 datasets followed by examples of 24h rhythmic/arrhythmic gene/transcript (lower panel). Circadian rhythmic genes/transcripts were plotted using harmonic regression fit and arrhythmic genes/transcripts were plotted using Loess fit in R. Frequency bar plots depict **b** the number of expressed transcripts per expressed gene, **c** the number of expressed transcripts per 24h rhythmic gene in HCT116 datasets. **d** Box plots depict RNA-seq data in the KO compared to WT cell lines. *** $p < 0.001$, (n = 16; t-test).

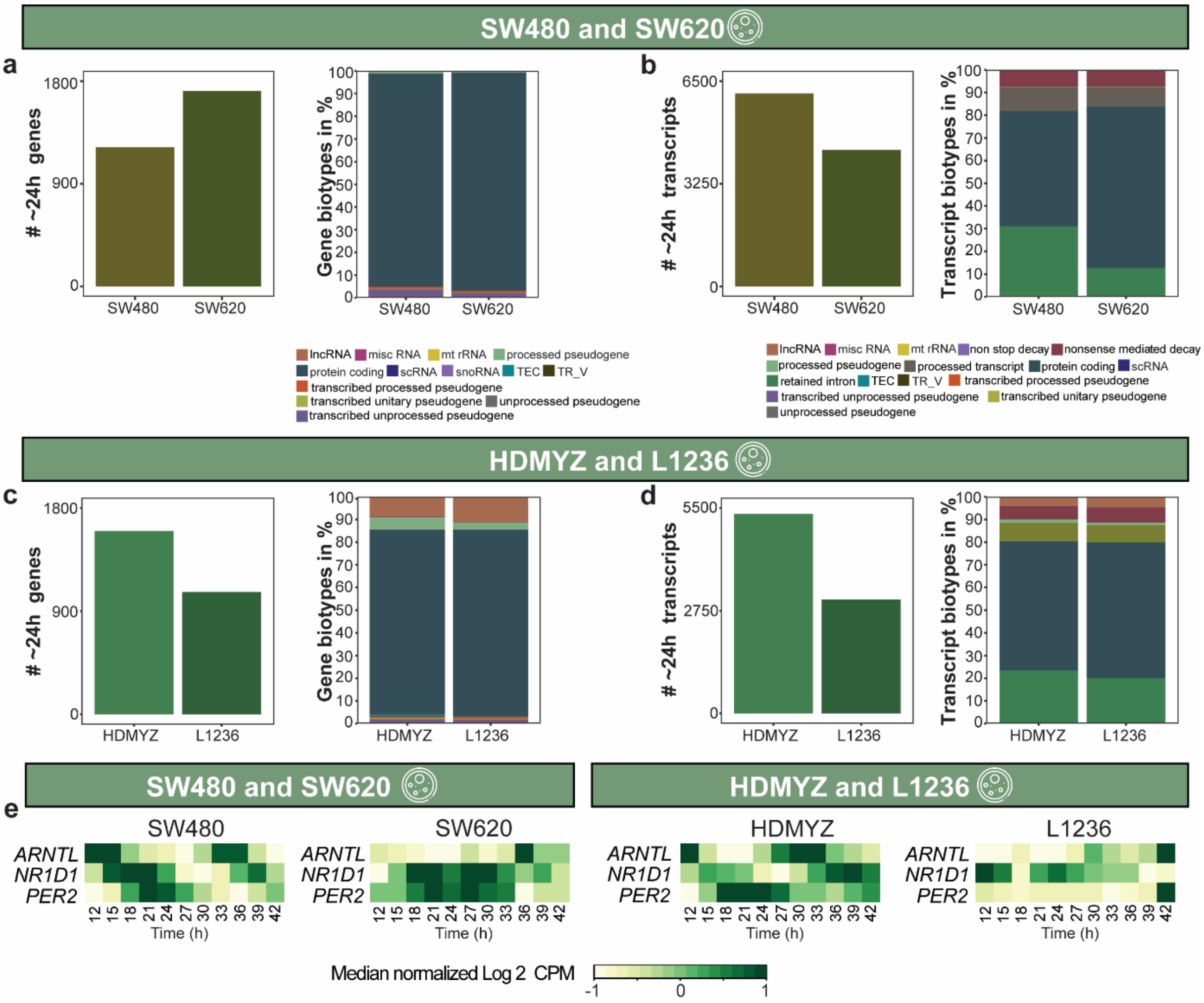


**Supplementary Figure 2: Classification of 24h rhythmic features at gene-level and transcript-level and core clock gene expression in publicly available CRC and HL cell line datasets. a-b** Bar graphs represent the total number of ~24h genes/transcripts (left panel) and their biotypes in percent (right panel) identified in CRC cell lines (SW480, SW620). **c-d** Bar graphs represent the total number of ~24h genes/transcripts (left panel) and their biotypes in percent (right panel) identified in HL cell lines (HDMYZ, L1236). **e** Heatmaps depict the expression change of *ARNTL*, *PER2*, and *NR1D1* among SW480, SW620, HDMYZ, and L1236 cells.

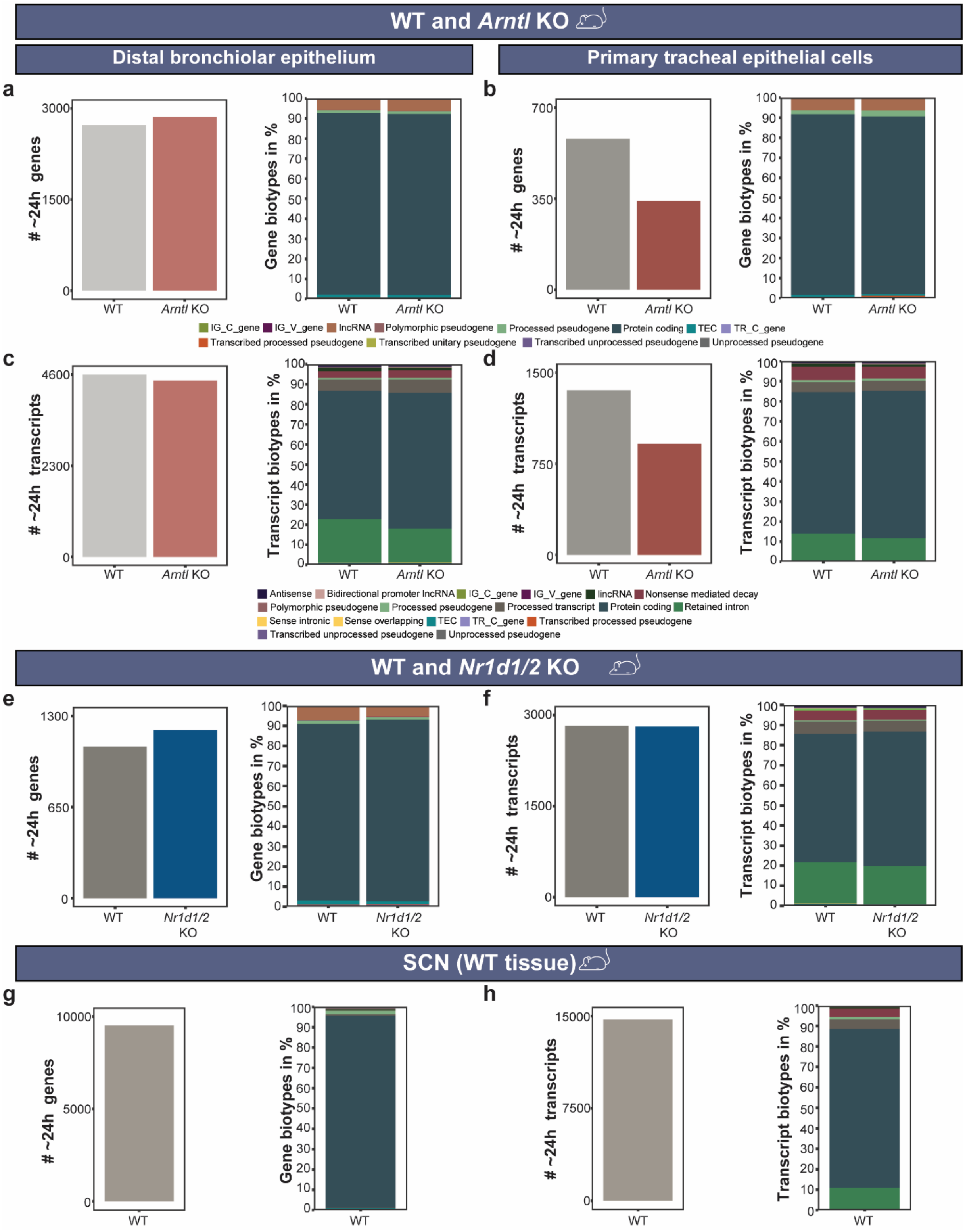


**Supplementary Figure 3: Classification of 24h rhythmic features at gene-level and transcript-level in murine RNA-seq datasets.** Bar graphs (left panel) represent the total number of ~24h genes/transcripts and their biotypes in percent (right panel) identified in **a, c** WT and *Arntl* KO murine model from distal bronchiolar epithelium tissue, **b, d** WT and *Arntl* KO murine model from primary tracheal epithelial cells, **e-f** WT and *Nr1d1/2* KO from epithelial cells, and **g-h** WT SCN tissue.

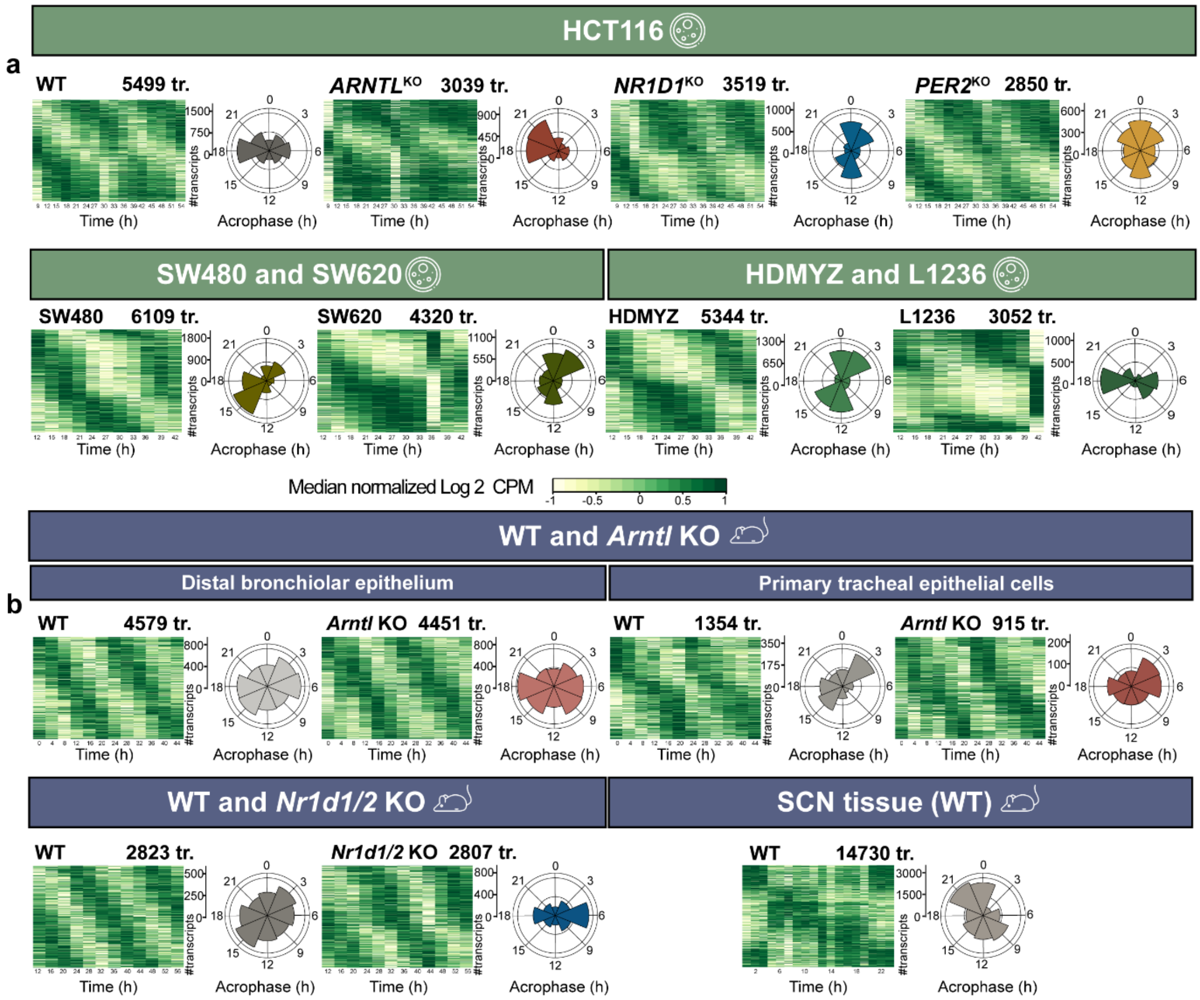


**Supplementary Figure 4: Median normalized phase-sorted heatmaps and acrophase bin visualization of 24h rhythmic transcripts in human and murine RNA-seq datasets.** Decline in ~24h transcripts (tr.) and changes in acrophase were observed upon KO for core clock genes in **a** human circadian datasets of HCT116$^{KO}$ vs. WT (upper panel), SW620 vs. SW480 (lower left panel), L1236 vs. HDMYZ (lower right panel), and in **b** murine circadian datasets of *Arntl* KO vs. WT from distal bronchiolar epithelium (upper left panel) & primary tracheal epithelial cells (upper right panel), *Nr1d1/2* KO vs. WT from epithelial cells (lower left panel), and SCN WT tissue (lower right panel).

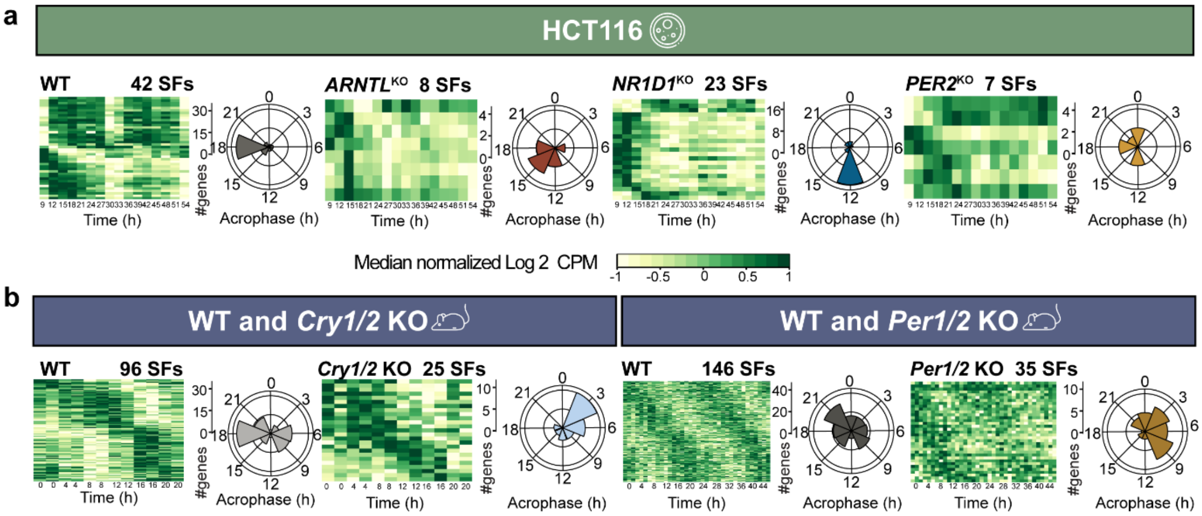


**Supplementary Figure 5: Clock disruption affects the rhythmic properties of splicing factors.** 24h rhythmic genes were mapped to the list of curated SFs to obtain the list of rhythmic splicing factors. Median normalized phase-sorted heatmaps and acrophase bin plots were generated to visualize 24h rhythmic splicing factors in HCT116 and murine *Cry1/2* KO and *Per1/2* KO datasets. **a** Decline in the number of 24h rhythmic SF genes and changes in acrophase bins were observed in HCT116 *ARNTL*, *NR1D1*, and *PER2* KO vs. WT. **b** *Cry1/2* KO resulted in a decline in the rhythmic SFs genes vs WT (left panel). Discrepancies in acrophase bin distribution of SFs was clearly visible in *Cry1/2* KO mouse vs WT. Disruption of *Per1/2* in mouse resulted in loss of rhythmic SFs when compared to its WT (right panel).

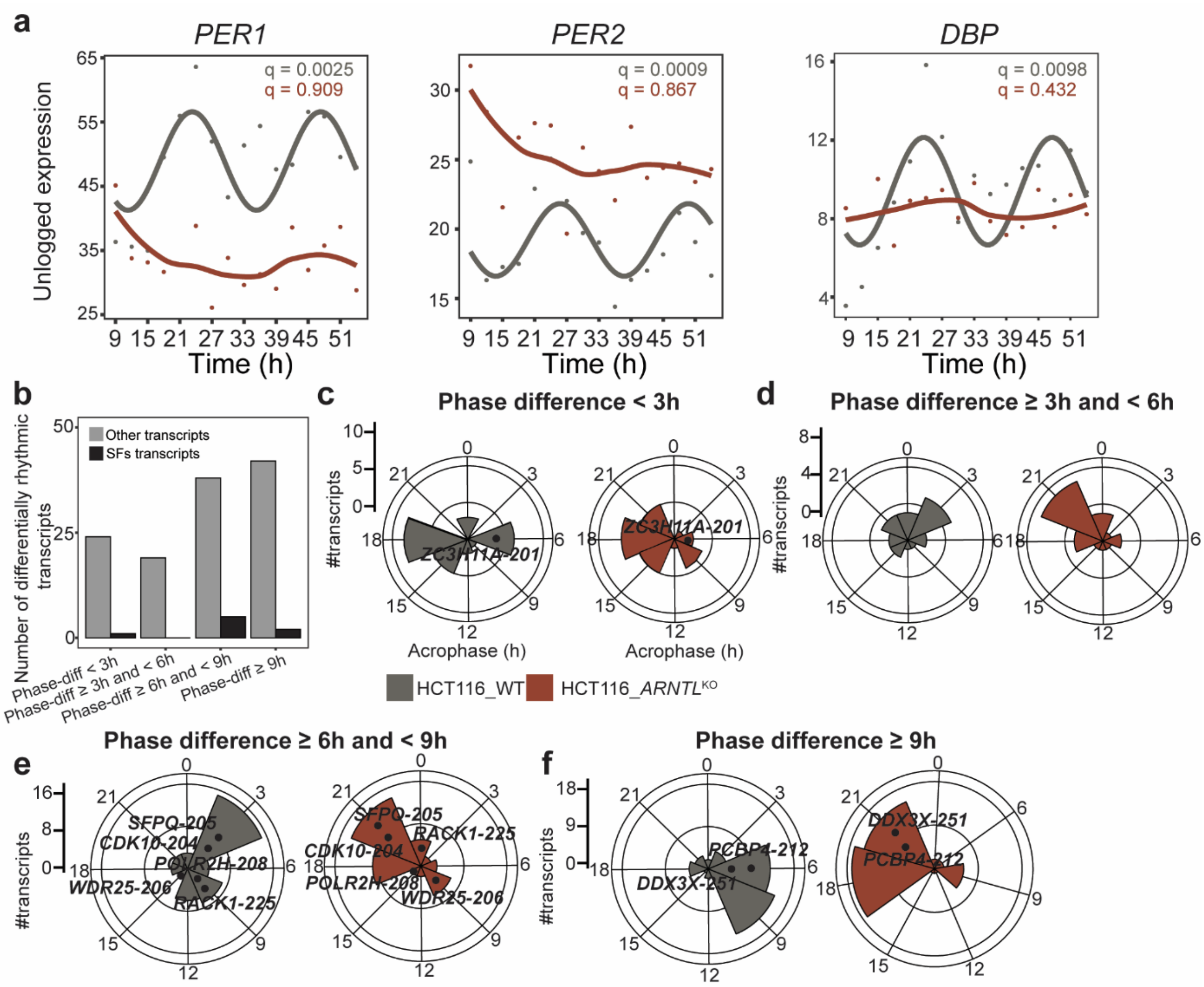


**Supplementary Figure 6: *ARNTL* disruption in HCT116 cells results in loss of rhythmicity in core clock elements and phase shift of several transcripts. a** *ARNTL* disruption in HCT116 cells resulted in the loss of rhythmicity in *PER1*, *PER2* and *DBP* genes. Circadian rhythmic genes were plotted using harmonic regression fit and arrhythmic genes were plotted using Loess fit in R. **b** Box plot depicts a higher number of transcripts with phase shift greater than 9h and a higher number of SF transcripts with phase shift between 6h and 9h. Acrophase bin plots represent the distribution of other transcripts along with SF transcripts (highlighted in black) with **c** Phase difference < 3h, **d** Phase difference between 3h and 6h, **e** Phase difference between 6h and 9h, and **f** Phase difference greater than 9h.

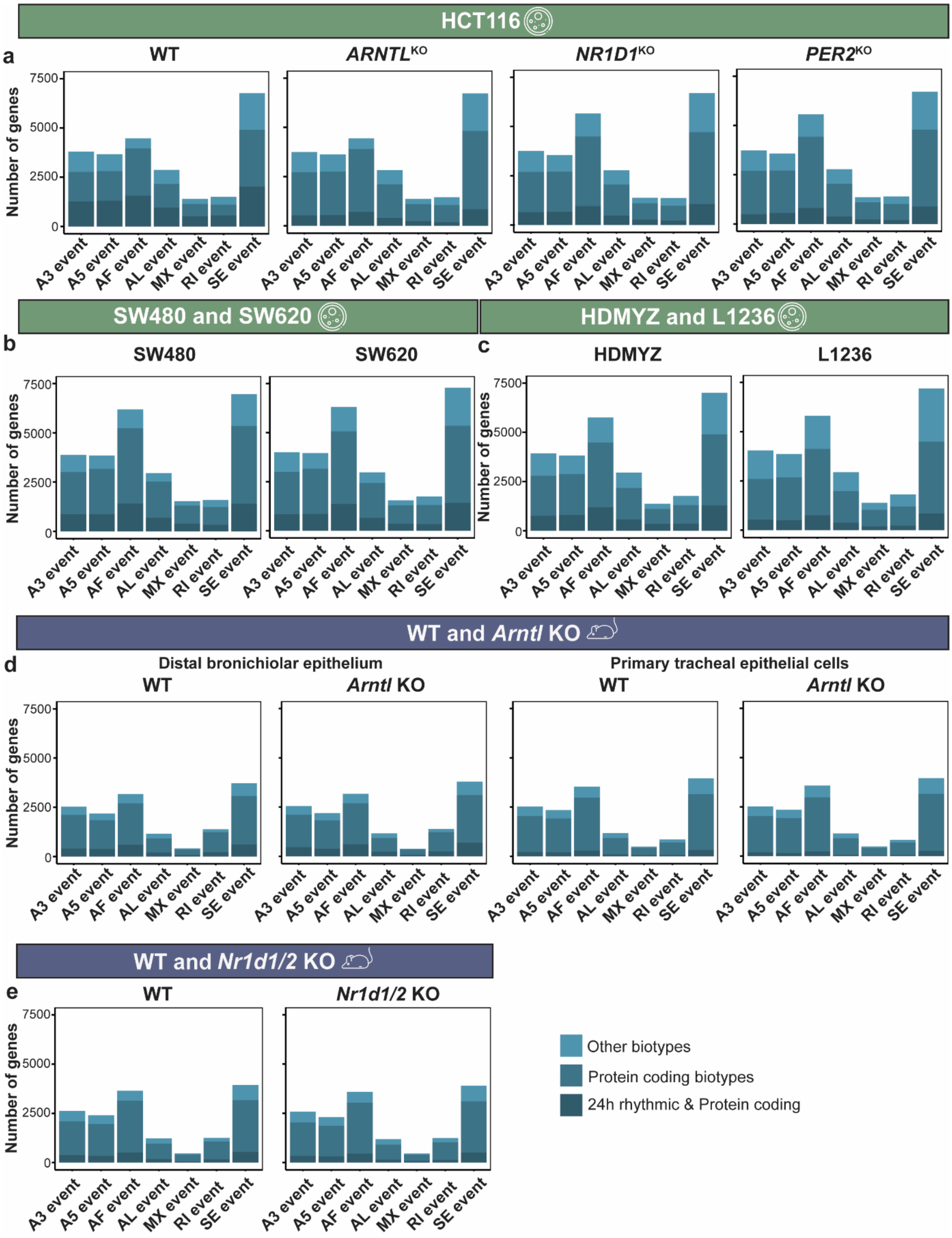


**Supplementary Figure 7: Local alternative splicing analysis revealed overall changes in total number of genes being alternatively spliced by either of seven modes in human and murine RNA-seq datasets.** Genes with significant splicing events were obtained (0.1<PSI< 0.9). Bar plots depict the total number of genes (x-axis) whose transcripts were alternatively spliced with the mentioned local splicing event (y-axis) in human cell lines datasets **a** HCT116_WT and KO, **b** SW480 & SW620, **c** HDMYZ & L1236 and in murine datasets **d** WT and *Arntl* KO, & **e** WT and *Nr1d1/2* KO. The number of genes containing transcripts with circadian expression and protein-coding biotype (dark blue), protein-coding biotype (blue), and different biotypes (light blue) in the corresponding AS groups are indicated.

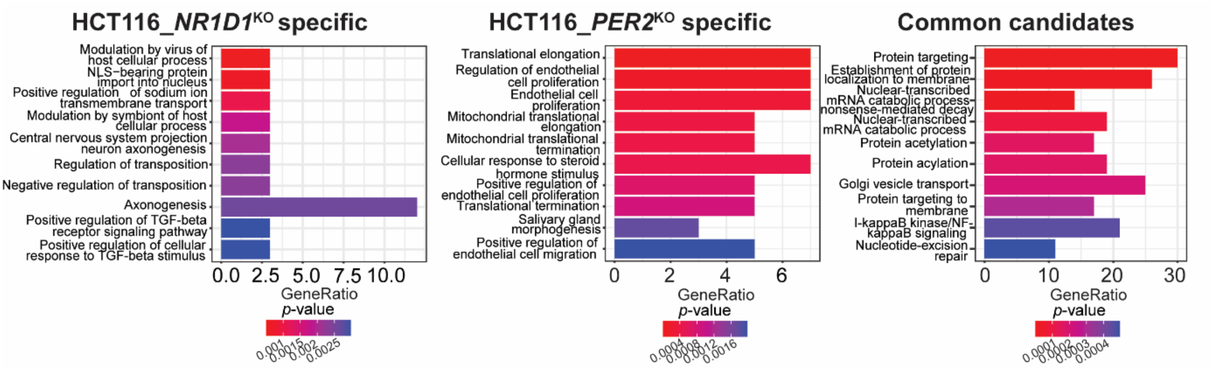


**Supplementary Figure 8: Functional enrichment analysis of unique and common candidates with AF-event gain in HCT116 *NR1D1* and *PER2* knockout cells.** To investigate the biological role of AF-event (gain) candidates, we shortlisted the genes with at least one protein-coding transcript and carried out a functional enrichment analysis. *NR1D1* specific candidates were enriched in processes like TGF-beta regulation (left panel), and *PER2* specific candidates were enriched in processes such as endothelial cell proliferation (middle panel). Common AF-event gain candidates between *PER2* and *NR1D1* KO were enriched in processes like protein acetylation (right panel).

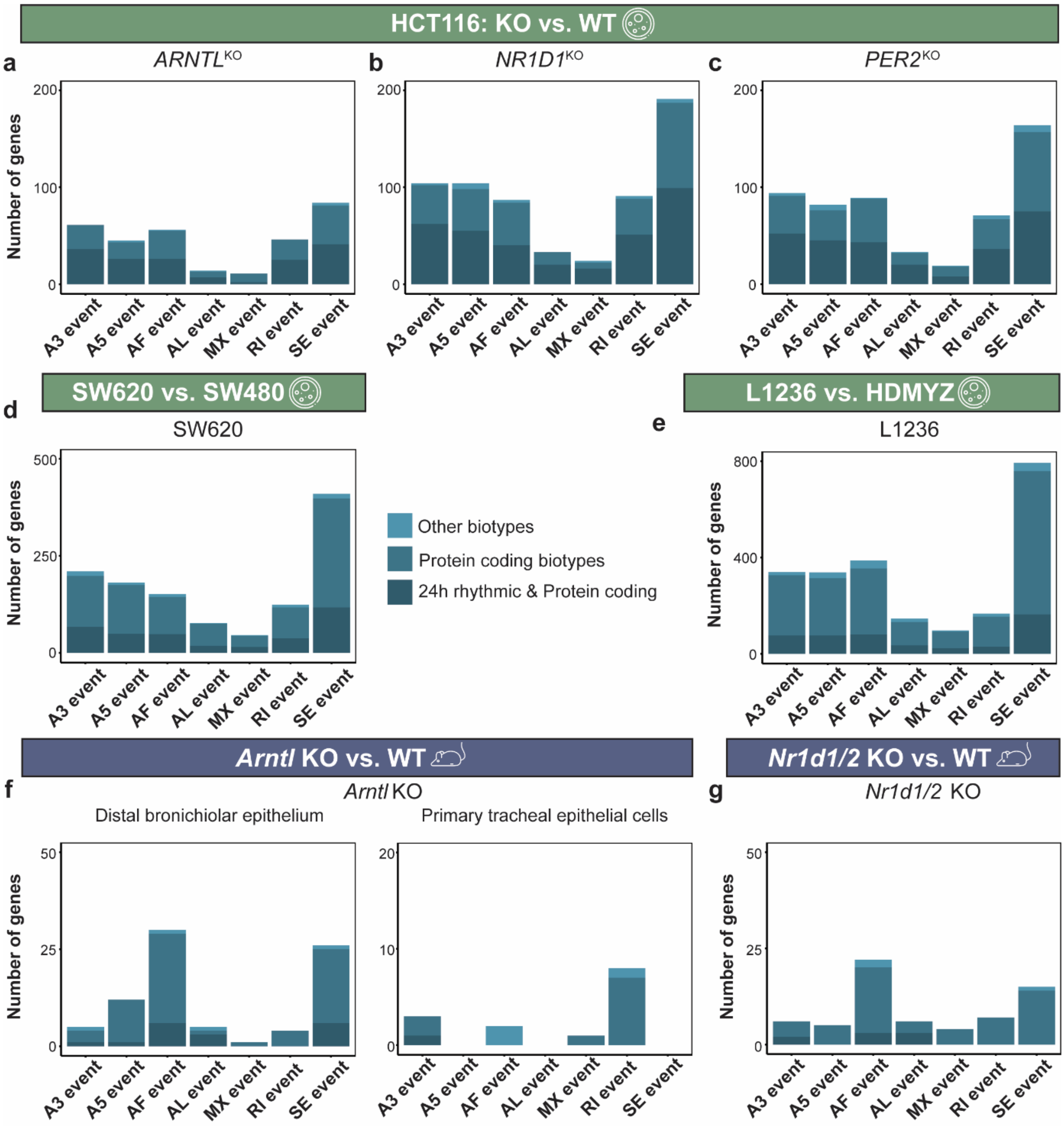


**Supplementary Figure 9: Differential splicing analysis showed higher number of significantly spliced genes with skipping exon event in human RNA-seq datasets.** Significant differentially spliced candidates were obtained ($p < 0.05$). Bar plots depict the total number of genes (x-axis) whose transcripts are differentially spliced when compared to their control group. The number of genes containing transcripts with circadian expression and protein-coding biotype (dark blue), protein-coding biotype (blue), and different biotypes (light blue) in the corresponding AS groups are indicated.

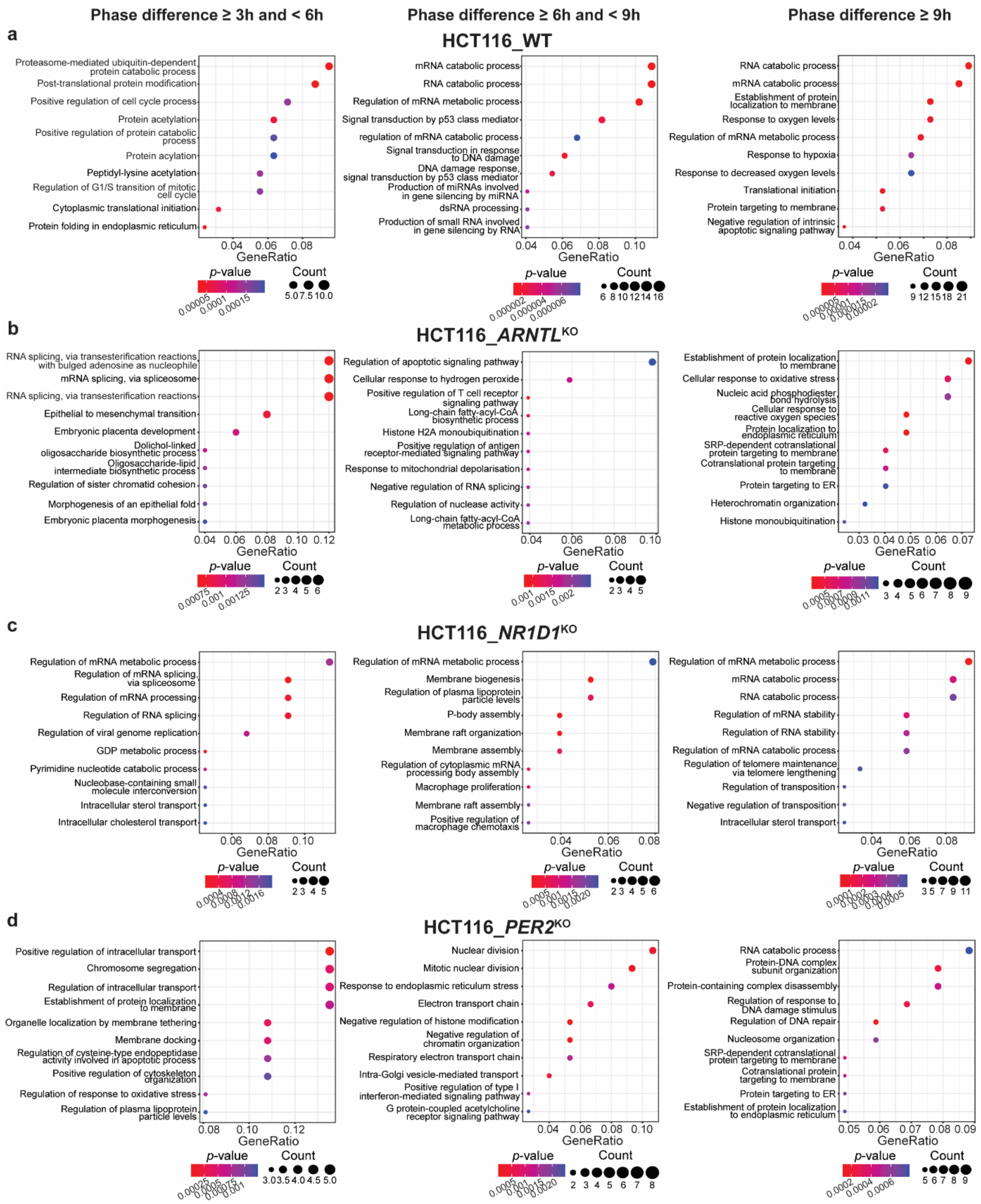


**Supplementary Figure 10: Functional enrichment analysis of genes with phase-shifted differentially rhythmic transcript pairs in HCT116 datasets.** To examine the enriched GO terms (biological processes) within phase-shifted transcript pairs, we grouped the genes based on a phase-difference ≥3h and <6h (left panel), phase-difference ≥6h and <9h (middle panel), and phase-difference ≥9h (right panel). **a** HCT116_WT, **b** HCT116_*ARNTL*$^{KO}$, **c** HCT116_*NR1D1*$^{KO}$, and **d** HCT116_*PER2*$^{KO}$.
